# Schisandrin B Inhibits Osteoclastogenesis and Protects Against Ovariectomy-Induced Bone Loss

**DOI:** 10.3389/fphar.2020.01175

**Published:** 2020-07-31

**Authors:** Jia Wang, Zhong Fang, Chao Song, Honglei Kang, Qian Guo, Yimin Dong, Ya Zhang, Renpeng Peng, Hanfeng Guan, Feng Li

**Affiliations:** Department of Orthopedics, Tongji Hospital, Tongji Medical College, Huazhong University of Science and Technology, Wuhan, China

**Keywords:** Schisandrin B, osteoclast, osteoporosis, MAPK, NF-κB, Nrf2

## Abstract

Osteoporosis is a systemic skeletal disease which is highly prevalent worldwide and considered to be associated with excessive bone resorption mediated by osteoclast. Osteoclast differentiation is featured by the activation of inflammation-related pathways and the generation of reactive oxygen species. Schisandrin B is a bioactive compound with strong antiinflammation and antioxidative properties, we thus speculated that Schisandrin B might serve as a potential candidate for osteoporosis. In the present study, we found that the formation and` function of osteoclasts were dramatically suppressed by Schisandrin B. And consistent with the *in vitro* results, treatment with Schisandrin B attenuated ovariectomy-induced bone loss in mice. Moreover, Schisandrin B notably inhibited the activation of mitogen activated protein kinase (MAPK) and nuclear factor-κB (NF-κB) pathways and scavenged ROS by activating nuclear factor E2 p45-related factor 2 (Nrf2) signaling. In conclusion, our study indicates that Schisandrin B is an effective approach to treat osteoporosis and other osteoclast-related diseases.

## Introduction

The homeostasis of bone is maintained by the cooperative effects of two types of cells: osteoblasts for bone formation and osteoclasts for bone resorption (Boyle et al., 2003). Imbalance of bone remodeling caused by an excessive number or overactive function of osteoclasts will eventually lead to many bone disorders, such as osteoporosis (Rodan and Martin, 2000). Osteoporosis is a common skeletal disease featured by low bone density and increased fracture risk, and it affects about 200 million globally, leaving behind serious social and economic problems (Pisani et al., 2016; Compston et al., 2019). However, the available treatments for osteoporosis are far from satisfactory (Weaver et al., 2010). Therefore, the identification of novel agents or mechanisms targeting osteoclast differentiation and function has substantial clinical implications.

Osteoclasts are multinucleated cells originating from the monocyte/macrophage lineage regulated by the collaborative effects of macrophage colony stimulating factor (M-CSF) and receptor activator of nuclear factor-κB ligand (RANKL) (Asagiri and Takayanagi, 2007). M-CSF maintains the survival and proliferation of osteoclasts (Ross and Teitelbaum, 2005), whereas RANKL provides signals essential for osteoclast precursor cells to differentiate into mature osteoclasts (Arai et al., 1999).

Previous studies have shed light on the multiple signaling pathways involved in RANKL-induced osteoclastogenesis. During osteoclast differentiation, MAPK and NF-κB signaling pathways are activated by the tumor necrosis factor receptor associated factor 6 (TRAF6), which is recruited *via* the interaction of RANKL and its receptor RANK on the surface of osteoclasts (Kaji et al., 2001; Novack, 2011). Additionally, stimulation by RANKL also generates reactive oxygen species (ROS), which are crucial for osteoclast differentiation and bone resorption reportedly (Garrett et al., 1990; Lee et al., 2005). Taken together, all these signaling cascades mentioned above participate in the regulation of osteoclastogenesis and contribute to the pathogenesis of osteoporosis and other osteoclast-related disorders.

Schisandrin B is a bioactive component isolated from the traditional Chinese medicine named Schisandra chinensis. Schisandrin B has shown varieties of biological and pharmacological properties, including antiinflammation and antioxidative activities (Ding et al., 2018; You et al., 2019). For instance, Schisandrin B has been reported to ameliorate chondrocytes inflammation and osteoarthritis by repression of MAPK and NF-κB pathways (Ran et al., 2018), indicating the potential mechanism of its antiinflammation property. In addition, a recent study revealed that Schisandrin B prevented diabetic nephropathy through suppressing oxidative stress (Mou et al., 2019). However, the precise role of Schisandrin B during RANKL-induced osteoclastogenesis remains obscure. In the current study, the role and the potential molecular mechanisms of Schisandrin B in osteoclast differentiation and function were investigated. Moreover, an estrogen deficiency-induced bone loss murine model was utilized to confirm the therapeutic effects of Schisandrin B on postmenopausal osteoporosis in mice.

## Materials and Methods

### Reagents

Purified Schisandrin B (purity: 99.99%), extracted from Schisandra chinensis, was obtained from MedChemExpress (Monmouth Junction, NJ, USA). Recombinant murine M-CSF and RANKL were purchased from R&D Systems (Minneapolis, MN, USA). Antibodies against NFATc1, c-FOS, P38, JNK, ERK, P65, IκBα, p-P38, p-JNK, p-ERK, p-P65, and p-IκBα were obtained from Cell Signaling Technology (Beverly, MA, USA). Antibody targeting TRAP was purchased from Abcam (Cambridge, MA, USA). Antibodies against MMP9, Nrf2, HO1, NQO1, and β-Actin were from Proteintech Group (Wuhan, Hubei, China). Trap staining kit and other reagents were purchased from Sigma-Aldrich (St. Louis, MO, USA).

### Bone Marrow Macrophages (BMMs) Preparation and Osteoclastogenesis Assay

Primary BMMs were obtained from bone marrow aspirates of 8-week-old C57BL/6 mice. In Brief, cells isolated from the femoral and tibial bone marrow were cultured in full α-MEM supplemented with 10% fetal bovine serum, 100 U/ml penicillin, 100 μg/ml streptomycin, and 30 ng/ml M-CSF. After 16 h, the supernatant cells were collected and maintained in the medium mentioned above for 2 days. The cells adhering to the bottom of the dishes were regarded as primary BMMs.

To generate osteoclasts, BMMs were further cultured in osteoclastogenic medium (α-MEM medium containing 30 ng/ml M-CSF and 75 ng/ml RANKL) for 5 days. Osteoclasts were then observed and analyzed using TRAP stanning kit according to the manufacturer’s protocols.

### Bone Marrow Stem Cells (BMSCs) Isolation and Osteoblast Differentiation Assay

Primary BMSCs were flushed out from bone marrow aspirates of 4-week-old C57BL/6 mice and cultured with basic α-MEM medium until confluent. Then the BMSCs were digested and seeded in 24-well plates at a density of 1×10^4^ cells/well. For osteoblast differentiation, the BMSCs were cultured in osteogenic medium (a-MEM medium containing 50 μM Ascorbic acid, 10 nM dexamethasone and 10 mM b-glycerol phosphate). ALP staining was performed on Day 7 and Alizarin Red staining was conducted on Day 21.

### Cell Viability Assay

BMMs were seeded in 96-well plates at a density of 5×10^3^ cells/well and incubated overnight to adhere. The cells were then treated with different concentrations of Schisandrin B for the indicated times. The cytotoxicity of Schisandrin B was measured by replacing the culture medium with fresh medium containing 10% CCK 8 solution (Boster Biotechnology, Wuhan, Hubei, China) and incubating at 37°C for 1 h. The optical density at 450 nm was detected by a microplate reader (Bio-Tek, Winooski, VT, USA).

### Pit Formation Assay

BMMs were seeded in an osteo assay surface coated with hydroxyapatite in a 96-well plate (Corning, NY, USA) at a density of 20,000 cells/well. Then the cells were cultured in osteoclastogenic medium for 5 days. After mature osteoclasts formed, various concentrations of Schisandrin B were incubated with osteoclasts. After 2 days, the plate was washed with 5% sodium hypochlorite and pit formation was quantitatively calculated through the resorption area.

### F-Actin Ring Assay

BMMs-derived osteoclasts cultured as previously described were fixed with immunol staining solution (Beyotime, Jiangsu, China) for 15 min and then permeabilized with 0.1% Triton X-100 for 5 min. Next, the cells were incubated with rhodamine‐conjugated phalloidin (Sigma-Aldrich) for 1 h at room temperature to visualize F‐actin. After staining with phalloidin, the nuclei were counterstained with DAPI for additional 5 min. F-actin rings were captured by a fluorescence microscope and total numbers per well were counted.

### Adenoviral Transduction

Adenoviruses carrying shRNA-targeting murine Nrf2 and control adenoviruses were purchased from Vigene Biosciences (Rockville, MD, USA). The shRNA sequence was as follows: 5′-CCGGCTTGAAGTCTTCAGCATGTTACTCGAGTAACATGCTGAAGACTTCAAGTTTTTT-3′.

For adenoviral transduction, BMMs were isolated as previously described and incubated in α-MEM medium with M-CSF supplementation. After 24 h, cells were washed and then cultured with adenoviral particles (100 particles per cell) for 12 h. Knockdown effects were confirmed by western blot.

### Murine Ovariectomy (OVX)-Induced Bone Loss Model

12-week-old female C57BL/6 mice were obtained from the Animal Center of Tongji Hospital (Wuhan, China) and all animal studies were approved by the Ethics Committee of Tongji Hospital. 40 healthy mice were randomly distributed to four groups (n = 8): SHAM+VEH group (sham operation and vehicle treatment), SHAM+Sch B group (sham operation and Schisandrin B treatment), OVX+VEH group (OVX operation and vehicle treatment), and OVX+Sch B group (OVX operation and Schisandrin B treatment, 30 mg/kg). The dosage used in mice was based on a previous study and our preliminary experiments (Li J. et al., 2019). One week after the surgery, mice were treated with Schisandrin B or vehicle by intragastrical gavage every other day. After 6 weeks, the mice were sacrificed to separate femurs and tibias for subsequent microcomputed tomography (micro-CT) scanning and histomorphometric analysis.

### Micro-CT Scanning and Histologic Analysis

Femurs of each mouse were scanned using a Scanco vivaCT 40 micro-CT instrument (Scanco Medical, Bassersdorf, Switzerland). The scanning protocol was set at 100 kV, 98 μA, and 10 μm voxel size. Three-dimensional reconstruction results such as percentage bone volume/tissue volume (BV/TV), mean trabecular thickness (Tb.Th), mean trabecular space (Tb.Sp), and mean trabecular numbers (Tb.N) were measured using the reconstruction system provided with the micro-CT.

After micro-CT scanning, the femurs were fixed in 4% paraformaldehyde and decalcified with 10% EDTA. Finally, the paraffin-embedded bone was sectioned for TRAP staining.

### RNA Isolation and Quantitative PCR

Total RNA was isolated from cultured cells by using TRIzol reagent (Invitrogen, Carlsbad, CA, USA) according to the manufacturer’s instructions. One microgram of total RNA was synthesized using the RevertAid First Strand cDNA Synthesis Kit (Thermo Scientific, Waltham, MA, USA). Real-time PCR was performed using SYBR Green Master Mix (Invitrogen). The primer sets used were as shown below (F, forward; R, reverse): NFATc1, F 5′-CAACGCCCTGACCACCGATAG-3′ and R 5′-GGGAAGTCAGAAGTGGGTGGA-3′; c-FOS, F 5′-CCAGTCAAGAGCATCAGCAA-3′ and R 5′-AAGTAGTGCAGCCCGGAGTA-3′; TRAP, F 5′-TACCTGTGTGGACATGACC-3′ and R 5′-CAGATCCATAGTGAAACCGC-3′; CTSK, F 5′-TGTATAACGCCACGGCAAA-3′ and R 5′-GGTTCACATTATCACGGTCACA-3′; MMP9, F 5′-TCCAGTACCAAGACAAAGCCTA-3′ and R 5′-TTGCACTGCACGGTTGAA-3′; CTR, F 5′-TGCTGGCTGAGTGCAGAAACC-3′ and R 5′-GGCCTTCACAGCCTTCAGGTAC-3′; DC-STAMP, F 5′-AAAACCCTTGGGCTGTTCTT-3′ and R 5′-AATCATGGACGACTCCTTGG-3′; V-ATPase, F 5′-GCCTCAGGGGAAGGCCAGATCG-3′ and R 5′-GGCCACCTCTTCACTCCGGAA-3′; GAPDH, F 5′-TCATTGACCTCAACTACATG-3′ and R 5′-TCGCTCCTGGAAGATGGTGAT-3′, Runx2, F 5’-GACTGTGGTTACCGTCATGGC-3’ and R 5’-ACTTGGTTTTTCATAACAGCGGA-3’; Osx, F 5’-ATGGCGTCCTCTCTGCTTG-3’ and R 5’-TGAAAGGTCAGCGTATGGCTT-3’; Alp, F 5’-CACGGCCATCCTATATGGTAA-3’ and R 5’-GGGCCTGGTAGTTGTTGTGA-3’; Ocn, F 5’-CTGACCTCACAGATCCCAAGC-3’ and R 5’-TGGTCTGATAGCTCGTCACAAG.

### Western Blotting

BMMs were treated with radio immunoprecipitation assay (RIPA) supplemented with 1 mM phenylmethanesulfonyl fluoride to extract proteins. Proteins were resolved on 10% SDS-PAGE gels, and the separated proteins were transferred onto PVDF membranes (Millipore, Billerica, MA, USA). After blocking with 5% BSA in TBS-0.1% Tween for 60 min, the membranes were incubated with indicated primary antibodies at 4°C overnight and secondary antibodies at 25°C for 60 min with gentle shaking. Antibody reactivity was detected by using electrochemical luminescence reagent (Thermo Scientific).

### Measurement of ROS Level

The ROS level was determined by using a ROS assay kit (Beyotime) in accordance with the manufacturer’s protocols. In brief, BMMs were incubated with various concentrations of Schisandrin B and stimulated with RANKL for 48 h. Subsequently, the cells were incubated with diluted DCFH-DA for 20min at 37°C in the dark and observed by a fluorescence microscope.

### Statistical Analysis

Data are presented as means ± SD of three independent experiments. Statistical differences were analyzed by one-way ANOVA or Student’s *t*-test, followed by Tukey’s *post hoc* analyses. *P*<0.05 was considered statistically significant.

## Results

### Schisandrin B Inhibits Osteoclast Differentiation

**Figure 1:**
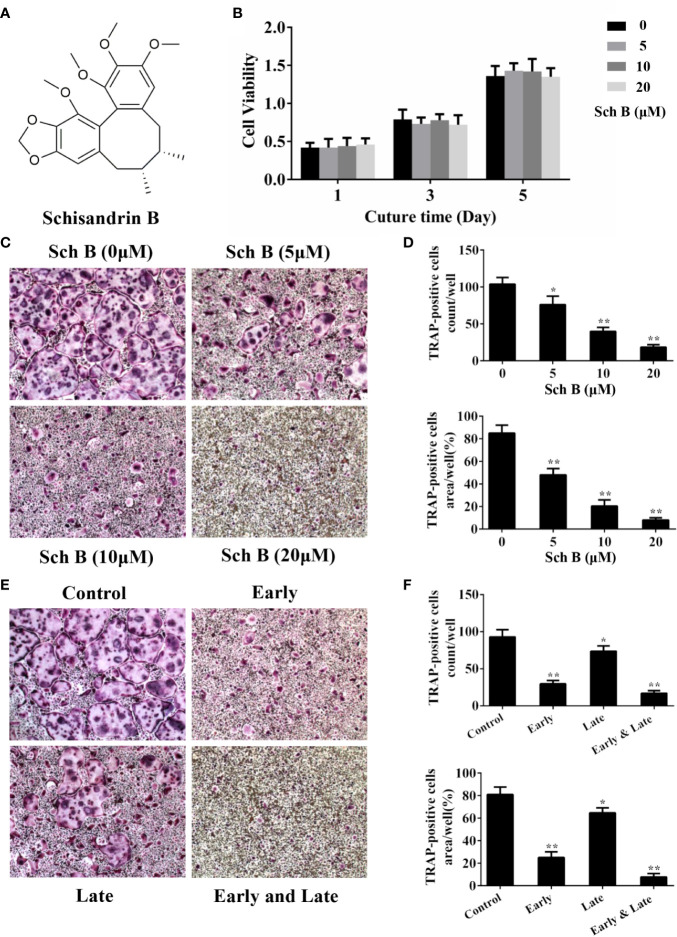
Schisandrin B inhibits osteoclast differentiation. **(A)** The chemical structure of Schisandrin B. **(B)** Bone marrow macrophages (BMMs) were treated with different concentrations of Schisandrin B (0, 5, 10, and 20 μM) for the indicated periods of time (1, 3, and 5 days). The cell viability was detected using CCK8 assay. **(C, D)** BMMs were treated with the indicated concentrations of Schisandrin B in the presence of macrophage colony stimulating factor (M-CSF) and receptor activator of nuclear factor-κB ligand (RANKL) for 5 days. TRAP-positive multinucleated osteoclasts were counted in the right column. **(E, F)** BMMs were treated with 20 μM Schisandrin B at early (1–3 days), late (3–5 days), and whole stages (1–5 days) of osteoclast differentiation in the presence of M-CSF and RANKL. TRAP-positive multinucleated osteoclasts were counted in the right column. The data were confirmed by three independent experiments and are expressed as means ± SD. *P < 0.05, **P < 0.01 versus the vehicle.

The chemical structure of Schisandrin B was shown in **Figure 1A**. To investigate the effects of Schisandrin B on osteoclastogenesis, the potential toxicity of Schisandrin B on BMMs was firstly measured. The CCK8 assay results revealed that the cell viability of BMMs treated with less than 20 μM Schisandrin B was not affected during RANKL-induced osteoclastogenesis (**Figure 1B**). Next, we evaluated whether Schisandrin B could inhibit osteoclast differentiation. The trap staining results showed the formation of osteoclasts was remarkably inhibited by Schisandrin B in a dose-dependent manner at 520 μM (**Figures 1C, D**). Osteoclast formation is a multistep process including proliferation, differentiation, fusion, and multinucleation. To test which stage was mainly suppressed by Schisandrin B, BMMs were treated with 20μM Schisandrin B at early (1–3 days), late (3–5 days), and whole stages (1–5 days) of osteoclast differentiation respectively. We found the inhibitory effects of Schisandrin B at the early stage of osteoclastogenesis were much stronger than those at the late stage (**Figures 1E, F**).

### Schisandrin B Suppresses Osteoclast Function

**Figure 2:**
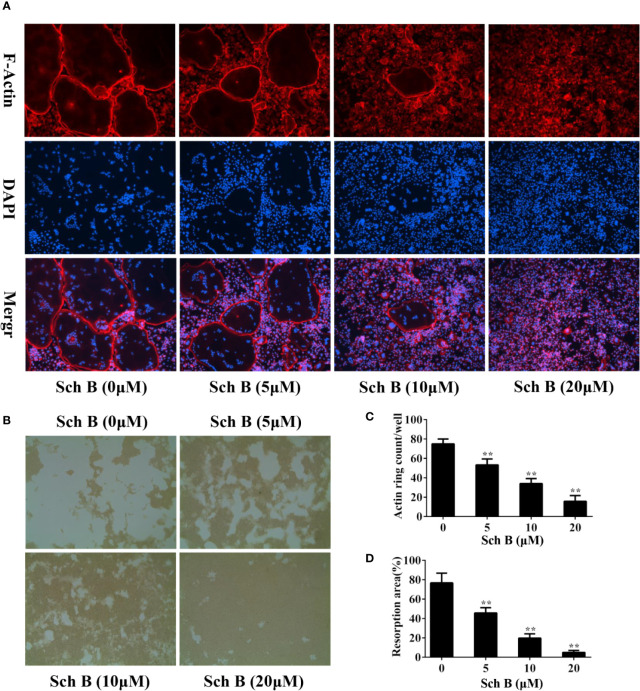
Schisandrin B suppresses osteoclast function. **(A, B)** Bone marrow macrophages (BMMs) were cultured in the presence of macrophage colony stimulating factor (M-CSF) and receptor activator of nuclear factor-κB ligand (RANKL) until mature osteoclasts formed, followed by treatment with the indicated concentrations of Schisandrin B for additional 2 days. F-actin staining assay **(A)** or pit formation assay **(B)** was performed. **(C, D)** F-Actin ring numbers and resorption pit areas were quantified. The data were confirmed by three independent experiments and are expressed as means ± SD. **P < 0.01 versus the vehicle.

To further explore whether Schisandrin B impaired osteoclast function, F-actin ring assay and pit formation assay were conducted. F-actin is a characteristic structure formed when osteoclasts attach to the bone surfaces and it is a prerequisite for osteoclastic bone resorption (Lakkakorpi and Vaananen, 1996; Zalli et al., 2016). As shown in **Figures 2A, C**, the numbers and the sizes of F‐actin rings were noticeably decreased by Schisandrin B treatment. Consistent with the results of F-actin ring assay, Schisandrin B also dramatically inhibited osteoclast-mediated resorption pits formation (**Figures 2B, D**).

### Schisandrin B Ameliorates OVX-Induced Bone Loss

**Figure 3:**
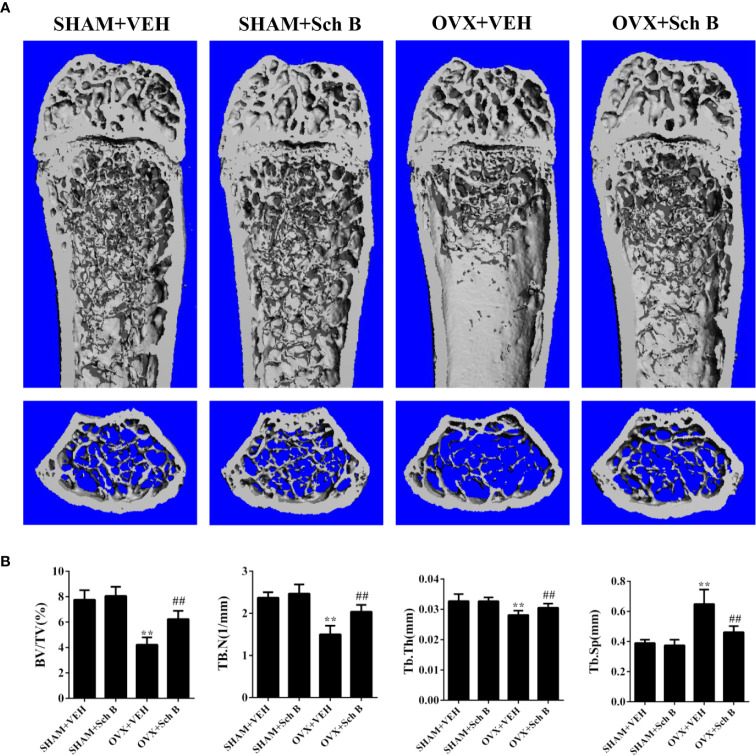
Schisandrin B attenuates ovariectomy-induced bone loss. **(A)** Representative micro-CT reconstruction results of the indicated four groups. **(B)** Micro-CT analyses of BV/TV, Tb.N, Tb.Th, and Tb.Sp in the region of interest. Data are presented as means ± SD. **P < 0.01 versus the SHAM+VEH group. ^##^*P* < 0.01 versus the OVX+VEH group.

**Figure 4:**
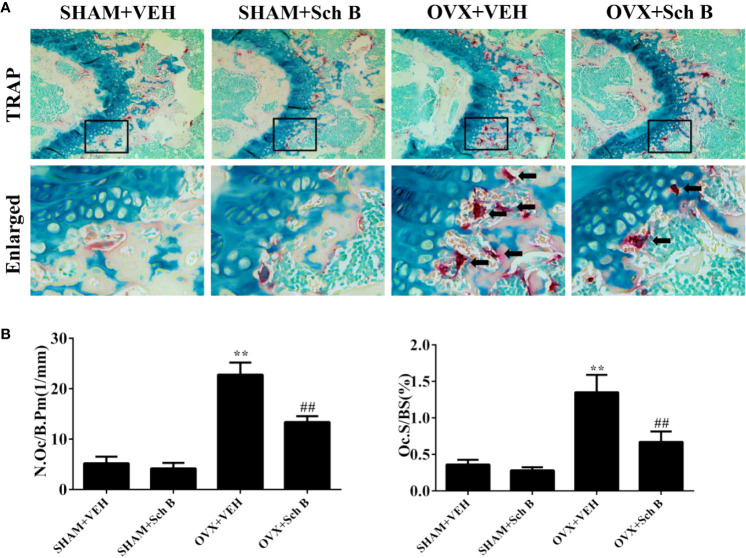
Schisandrin B reduces osteoclast formation in an ovariectomized murine model. **(A)** TRAP staining results of representative sections of distal femurs in the indicated four groups. **(B)** Bone histomorphometric analyses for number of osteoclasts per bone perimeter (N.Oc/B.Pm) and osteoclast surface per bone surface (Oc.S/BS). Data are presented as means ± SD. **P < 0.01 versus the SHAM+VEH group. ^##^*P* < 0.01 versus the OVX+VEH group.

A murine model of estrogen deficiency-induced bone loss was utilized to mimic postmenopausal osteoporosis and micro-CT scanning was performed on the distal femurs of each mice to evaluate bone mass changes. Our results showed the OVX mice experienced a substantial loss of trabecular bone compared with the sham-operated mice. Meanwhile, Schisandrin B treatment in the OVX mice effectively attenuated the trabecular bone loss (**Figure 3A**). Additionally, the OVX mice treated with Schisandrin B exhibited an increase in BV/TV, Tb.N, and Tb.Th, and a decrease in Tb.Sp in comparison to the mice receiving vehicle treatment (**Figure 3B**). Next, TRAP staining was conducted to determine whether Schisandrin B protected against estrogen deficiency-induced bone loss by suppressing osteoclast differentiation. The TRAP-stained sections indicated that Schisandrin B reduced the numbers of TRAP-positive cells which were increased by estrogen deficiency (**Figures 4A, B**).

### Schisandrin B Downregulates Osteoclast-Related Genes Expression

**Figure 5:**
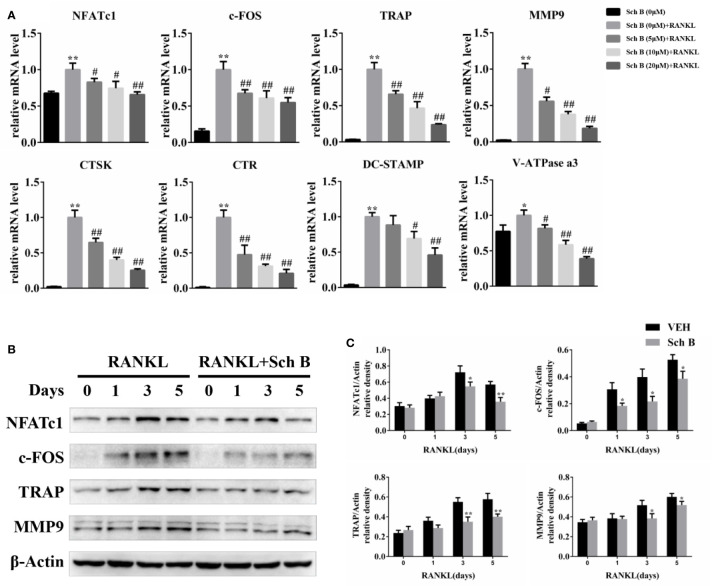
Schisandrin B downregulates osteoclast-related genes expression. **(A)** Bone marrow macrophages (BMMs) were treated with different concentrations of Schisandrin B (0, 5, 10, and 20 μM) in the presence of macrophage colony stimulating factor (M-CSF) and receptor activator of nuclear factor-κB ligand (RANKL) for 2 days. mRNA expression of osteoclast-related genes was measured by qPCR. **(B, C)** BMMs were cultured with or without 20μM Schisandrin B in the presence of M-CSF and RANKL for the indicated time periods. Protein expression of osteoclast-related genes was quantified using β-Actin as an internal control. The data were confirmed by three independent experiments and are expressed as means ± SD. *P < 0.05, **P < 0.01 versus the vehicle. ^#^*P* < 0.05, ^##^*P* < 0.01 versus the Sch B (0 μM) + RANKL group.

To further elucidate the effects of Schisandrin B on osteoclast differentiation, the expression of osteoclast specific genes including NFATc1, c-FOS, TRAP, MMP9, CTSK, CTR, DC-STAMP, and V-ATPase a3 was measured. Quantitative PCR results revealed the mRNA levels of these genes were all downregulated by Schisandrin B treatment (**Figure 5A**). The inhibitory effects of Schisandrin B on osteoclast-related genes expression were further confirmed by western blot analysis, which showed that Schisandrin B dramatically suppressed the protein expression of these target genes during osteoclast differentiation (**Figures 5B, C**).

### Schisandrin B Represses RANKL-Induced NF-κB, JNK, and ERK Phosphorylation

**Figure 6:**
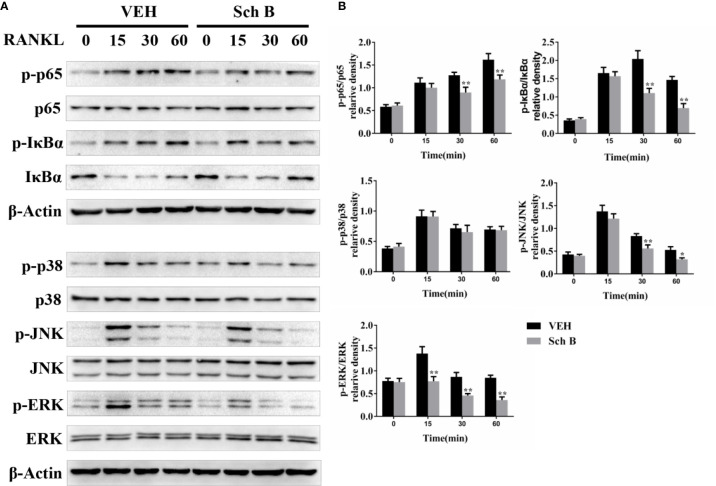
Schisandrin B represses receptor activator of nuclear factor-κB ligand (RANKL)–induced NF-κB and mitogen activated protein kinase (MAPK) activation. **(A, B)** BMMs were starved in 0.5% FBS complete medium for 12 h. Then the cells were pretreated with or without Schisandrin B (20μM) for 24 h. Before the collection of protein extracts, BMMs were stimulated with RANKL for 0, 15, 30, or 60 min. The components of NF-κB and MAPK signaling pathways were measured by Western blotting. The data were confirmed by three independent experiments and are expressed as means ± SD. *P < 0.05, **P < 0.01 versus the vehicle.

To investigate the mechanisms involved in Schisandrin B-mediated suppression of osteoclastogenesis, the effects of Schisandrin B on NF-κB and MAPK pathways were detected. As shown in **Figures 6A, B**, Schisandrin B markedly repressed NF-κB activation by inhibiting the degradation of IκBα and the phosphorylation of P65. As for the MAPK pathways, p-JNK and p-ERK were clearly decreased in Schisandrin B-treated BMMs, whereas the phosphorylation of P38 was not obviously affected by Schisandrin B treatment.

### Schisandrin B Inhibits Oxidative Stress and Activates Nrf2 Signaling

**Figure 7:**
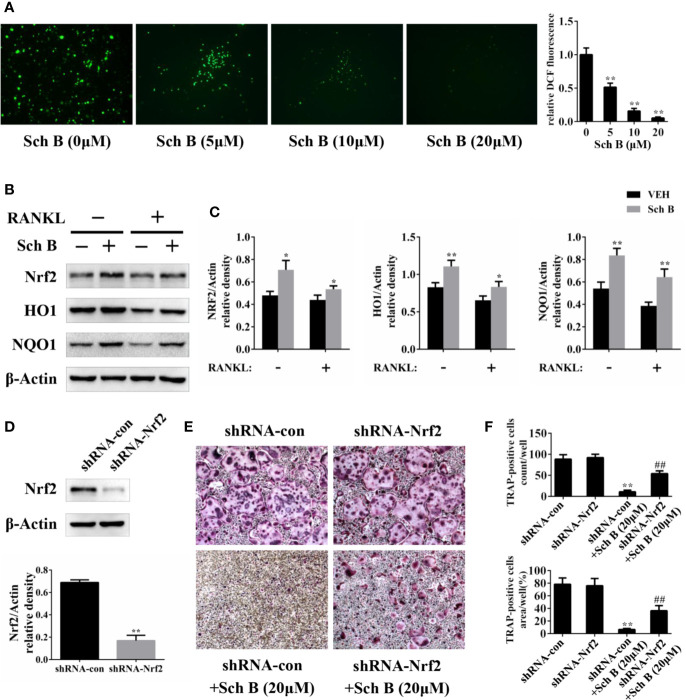
Schisandrin B inhibits oxidative stress and activates Nrf2 signaling. **(A)** Bone marrow macrophages (BMMs) were treated with the indicated concentrations of Schisandrin B in the presence of macrophage colony stimulating factor (M-CSF) and receptor activator of nuclear factor-κB ligand (RANKL) for 2 days. Cellular reactive oxygen species (ROS) levels were evaluated and quantified. **(B, C)** BMMs were treated with or without Schisandrin B (20μM) in the absence or presence of RANKL for 3 days. The protein was extracted for Western blotting. **(D)** Knockdown efficacy of adenoviral transduction. BMMs were infected with the adenovirus carrying Nrf2 shRNA or the control adenovirus for 3 days, and the expression of Nrf2 was measured using western blotting. **(E, F)** BMMs were treated with Schisandrin B (20μM), the adenovirus carrying Nrf2 shRNA, and the control adenovirus as indicated. After 5 days, TRAP staining was conducted. The data were confirmed by three independent experiments and are expressed as means ± SD. *P < 0.05, **P < 0.01 versus the vehicle. ^##^*P* < 0.01 versus the shRNA-con+ Schisandrin B (20μM) group.

Oxidative stress has been reported to play a vital role during osteoclast differentiation, so the influences of Schisandrin B on ROS production were evaluated. The generation of ROS induced by RANKL was strongly inhibited by Schisandrin B administration in a concentration-dependent manner (**Figure 7A**). Nrf2 is known to modulate oxidative stress through induction of multiple antioxidant enzymes. Western blotting results showed the protein levels of Nrf2 and its target genes such as HO1 and NQO1 were enhanced by Schisandrin B independent of RANKL stimulation (**Figures 7B, C**). To further confirm whether Schisandrin B inhibited osteoclastogenesis by activating Nrf2, a loss of function experiment was conducted. Firstly, knockdown effects of the adenovirus carrying Nrf2 shRNA were identified by western blot (**Figure 7D**). Then the trap staining results revealed that the inhibitory effects of Schisandrin B on osteoclast differentiation were partially reversed by the adenovirus carrying Nrf2 shRNA (**Figures 7E, F**).

## Discussion

Osteoporosis is a common bone disorder causing heavy burdens on global public health. Although great progress has been made in the treatment for osteoporosis, the toxic and side effects of current antiosteoporotic drugs cannot be ignored (Cappuzzo and Delafuente, 2004; Awasthi et al., 2018). Thus, it is necessary to develop effective and safe candidates to treat osteoporosis.

Schisandrin B, a major bioactive component of Schisandra chinensis, has strong antiinflammation and antioxidative properties (Ding et al., 2018; You et al., 2019). And it belongs to dibenzocyclooctadiene derivatives chemically. In general, Schisandrin B has shown various benefits in different organs and diseases, such as osteoarthritis, renal fibrosis, and myocardial ischemia (Ran et al., 2018; Zhao et al., 2018; Cao et al., 2019), and it has been reported to regulate several signaling pathways, including NF-κB, MAPK, PI3K/AKT, and Nrf2/HO-1 pathways (Zhu et al., 2017; Feng et al., 2018; Zhao et al., 2018; You et al., 2019).

In the present study, the effects of Schisandrin B on osteoclast *in vitro* and *in vivo* were investigated. We found that Schisandrin B significantly inhibited osteoclast differentiation and function. Additionally, it exhibited no cytotoxicity during RNAKL-induced osteoclastogenesis. Moreover, treatment with Schisandrin B dramatically attenuated bone loss in an estrogen deficiency murine model and no deaths or side effects were observed during the intervention process. Previous studies have shown that other substances with antiosteoclastic activity, such as flavonoids, terpenoids, and alkaloids can also attenuate ovariectomy-induced bone loss (Cai et al., 2018; Hu et al., 2019; Yin et al., 2019). Collectively, these results indicated Schisandrin B might be a promising method for treating osteoporosis and other osteoclast-related diseases.

Osteoclastic bone resorption and osteoblastic bone formation maintain bone homeostasis together, so some experiments on osteoblast were performed as a supplement. In the current study, we found Schisandrin B can promote osteoclast differentiation (**Supplementary Figure 1**), and these results were partially in agreement with previous studies (Caichompoo et al., 2009; Takanche et al., 2020). Osteoblasts are derived from mesenchymal stem cells, and besides osteoblasts, mesenchymal stem cells can also differentiate into other cell types, including adipocytes, myocytes, and chondrocytes (Jackson et al., 2012). A previous study showed that Gomisin N, extracted from Schisandra chinensis, reduced PPARγ expression and inhibited adipogenesis (Jang et al., 2017). Taken together, Schisandra chinensis showed osteoblastic and antiadipogenic activities reportedly.

MAPK and NF-κB are two crucial signaling cascades involved in RANKL-induced osteoclastogenesis (Franzoso et al., 1997; David et al., 2002; Li Z. et al., 2019). The binding of RANKL and its receptor RANK recruits TRAF6, eventually leading to the activation of MAPK and NF-κB pathways. The important role of Schisandrin B in the above pathways has been well established. For example, Schisandrin B was proved to ameliorate cardiac remodeling by suppressing MAPK signaling pathway (Ai et al., 2019). In addition, Schisandrin B was reported to improve the renal function of IgA nephropathy by repressing NF-κB pathway (Qin et al., 2019). Another study also showed Schisandrin B could attenuate vascular endothelial cells injury through regulation of NF-κB pathway (Lin et al., 2019). In the present study, the activation of MAPK and NF-κB signaling pathways was also inhibited by Schisandrin B, which is consistent with these previous studies. Our further investigation revealed that Schisandrin B suppressed the expression of NFATc1, c-FOS, TRAP, MMP9, CTSK, CTR, DC-STAMP, and V-ATPase, which are hallmarks of osteoclast differentiation.

ROS are reactive molecules formed from oxygen that contribute to various physiological and pathological conditions. They are produced by NADPH oxidase activation during RANKL-induced osteoclastogenesis and play essential roles of osteoclast formation (Steinbeck et al., 1994; Darden et al., 1996). Nrf2 is a transcription factor involved in osteoclast differentiation by reducing ROS *via* induction of antioxidant enzymes such as HO1 and NQO1 (Hyeon et al., 2013; Sun et al., 2015). The critical effects of Schisandrin B on scavenging ROS has been proved. For example, Schisandrin B was reported to protect against liver injury by attenuating oxidative stress (Deng et al., 2014). Moreover, Schisandrin B can prevent endotheliocyte deficits by activating Nrf2 signaling (Han et al., 2018). In the present study, ROS were scavenged by Schisandrin B in a dose-dependent manner. Meanwhile, Schisandrin B activated Nrf2 and promoted the expression of antioxidant enzymes, indicating that Schisandrin B suppressed osteoclastogenesis by eliminating ROS and activating Nrf2 signaling. Interestingly, we also found that the inhibitory effects of Schisandrin B on osteoclast differentiation were partially reversed by the adenovirus carrying Nrf2 shRNA, further elucidating the essential role of Nrf2 in Schisandrin B-mediated osteoclastogenesis repression.

Certain limitations of this study should be addressed despite the promising results. Schisandrin A, Schisandrin B, and Schisandrin C are the representative components of Schisandra chinensis. A previous study comparing Schisandrin A and Schisandrin B showed that only Schisandrin B resulted in the activation of Nrf2 (Leong et al., 2016), which is essential to osteoclastogenesis (Hyeon et al., 2013), so we selected Schisandrin B to investigate its role in osteoclast differentiation. And another study has also reported the inhibitory effect of Schisandra chinensis on osteoclastogenesis (Kim et al., 2018). Therefore, further studies investigating the differences and connections of the three components may help to better understand the exact function of Schisandra chinensis.

In conclusion, our study demonstrated Schisandrin B inhibited osteoclastogenesis *via* repression of MAPK and NF-κB pathways and scavenging of ROS *in vitro* and attenuated OVX-induced bone loss *in vivo*.

## Data Availability Statement

The datasets generated for this study are available on request to the corresponding authors.

## Ethics Statement

The animal study was reviewed and approved by the Ethics Committee of Tongji Hospital.

## Author Contributions

JW, ZF, HG, and FL designed the study. JW, HK, QG, and YD conducted the study. JW, CS, YZ, and RP analyzed the data. JW, HG, and FL wrote the manuscript.

## Funding

The study was supported in part by the National Key R&D Program of China (No. 2016YFB1101305) and the National Natural Science Foundation of China (No. 81874024).

## Conflict of Interest

The authors declare that the research was conducted in the absence of any commercial or financial relationships that could be construed as a potential conflict of interest.

## Supplementary Material

Supplementary Figure 1 The plant sample of Schisandrin B promotes osteoblast differentiation. BMSCs were cultured in osteogenic medium and treated with different concentrations of Schisandrin B as indicated. **(A)** representative Alizarin staining pictures, **(B)** representative ALP staining results, **(C)** mRNA levels of Runx2, Osx, Alp, and Ocn. *Plt;0.05, **P<0.01 versus the vehicle.

## References

[B1] Ai F. Guo Q. H. Yu B. Li W. Guo X. Chen Z. (2019). Schisandrin B attenuates pressure overload-induced cardiac remodeling in mice by inhibiting the MAPK signaling pathway. Exp. Ther. Med. 18 (6), 4645–4652. 10.3892/etm.2019.8154 31798701 PMC6878904

[B2] Arai F. Miyamoto T. Ohneda O. Inada T. Sudo T. Brasel K. (1999). Commitment and differentiation of osteoclast precursor cells by the sequential expression of c-Fms and receptor activator of nuclear factor kappaB (RANK) receptors. J. Exp. Med. 190 (12), 1741–1754. 10.1084/jem.190.12.1741 10601350 PMC2195707

[B3] Asagiri M. Takayanagi H. (2007). The molecular understanding of osteoclast differentiation. Bone 40 (2), 251–264. 10.1016/j.bone.2006.09.023 17098490

[B4] Awasthi H. Mani D. Singh D. Gupta A. (2018). The underlying pathophysiology and therapeutic approaches for osteoporosis. Med. Res. Rev. 38 (6), 2024–2057. 10.1002/med.21504 29733451

[B5] Boyle W. J. Simonet W. S. Lacey D. L. (2003). Osteoclast differentiation and activation. Nature 423 (6937), 337–342. 10.1038/nature01658 12748652

[B6] Cai C. Liu C. Zhao L. Liu H. Li W. Guan H. (2018). Effects of Taxifolin on Osteoclastogenesis in vitro and in vivo. Front. Pharmacol. 9:1286:1286. 10.3389/fphar.2018.01286 30483128 PMC6240596

[B7] Caichompoo W. Zhang Q. Y. Hou T. T. Gao H. J. Qin L. P. Zhou X. J. (2009). Optimization of extraction and purification of active fractions from Schisandra chinensis (Turcz.) and its osteoblastic proliferation stimulating activity. Phytother. Res. 23 (2), 289–292. 10.1002/ptr.2585 18698667

[B8] Cao G. Li S. Shi H. Yin P. Chen J. Li H. (2019). Schisandrin B attenuates renal fibrosis via miR-30e-mediated inhibition of EMT. Toxicol. Appl. Pharmacol. 385:114769. 10.1016/j.taap.2019.114769 31697999

[B9] Cappuzzo K. A. Delafuente J. C. (2004). Teriparatide for severe osteoporosis. Ann. Pharmacother. 38 (2), 294–302. 10.1345/aph.1D353 14742769

[B10] Compston J. E. McClung M. R. Leslie W. D. (2019). Osteoporosis. Lancet 393 (10169), 364–376. 10.1016/S0140-6736(18)32112-3 30696576

[B11] Darden A. G. Ries W. L. Wolf W. C. Rodriguiz R. M. Key L. L. Jr. (1996). Osteoclastic superoxide production and bone resorption: stimulation and inhibition by modulators of NADPH oxidase. J. Bone Miner. Res. 11 (5), 671–675. 10.1002/jbmr.5650110515 9157782

[B12] David J. P. Sabapathy K. Hoffmann O. Idarraga M. H. Wagner E. F. (2002). JNK1 modulates osteoclastogenesis through both c-Jun phosphorylation-dependent and -independent mechanisms. J. Cell Sci. 115 (Pt 22), 4317–4325. 10.1242/jcs.00082 12376563

[B13] Deng Y. Xu Z. Xu B. Liu W. Feng S. Yang T. (2014). Antioxidative effects of schidandrin B and green tea polyphenols against mercuric chloride-induced hepatotoxicity in rats. J. Environ. Pathol. Toxicol. Oncol. 33 (4), 349–361. 10.1615/jenvironpatholtoxicoloncol.2014011859 25404381

[B14] Ding M. Shu P. Gao S. Wang F. Gao Y. Chen Y. (2018). Schisandrin B protects human keratinocyte-derived HaCaT cells from tert-butyl hydroperoxide-induced oxidative damage through activating the Nrf2 signaling pathway. Int. J. Mol. Med. 42 (6), 3571–3581. 10.3892/ijmm.2018.3901 30272282

[B15] Feng S. Qiu B. Zou L. Liu K. Xu X. Zhu H. (2018). Schisandrin B elicits the Keap1-Nrf2 defense system via carbene reactive metabolite which is less harmful to mice liver. Drug Des. Devel. Ther. 12, 4033–4046. 10.2147/DDDT.S176561 PMC6267698 30568426

[B16] Franzoso G. Carlson L. Xing L. Poljak L. Shores E. W. Brown K. D. (1997). Requirement for NF-kappaB in osteoclast and B-cell development. Genes Dev. 11 (24), 3482–3496. 10.1101/gad.11.24.3482 9407039 PMC316809

[B17] Garrett I. R. Boyce B. F. Oreffo R. O. Bonewald L. Poser J. Mundy G. R. (1990). Oxygen-derived free radicals stimulate osteoclastic bone resorption in rodent bone in vitro and in vivo. J. Clin. Invest. 85 (3), 632–639. 10.1172/JCI114485 2312718 PMC296476

[B18] Han J. Shi X. Zheng Z. Zhang B. Shi F. Jiang L. (2018). Schisandrin B protects against angiotensin II-induced endotheliocyte deficits by targeting Keap1 and activating Nrf2 pathway. Drug Des. Devel. Ther. 12, 3985–3997. 10.2147/DDDT.S184245 PMC6255115 30538426

[B19] Hu B. Sun X. Yang Y. Ying Z. Meng J. Zhou C. (2019). Tomatidine suppresses osteoclastogenesis and mitigates estrogen deficiency-induced bone mass loss by modulating TRAF6-mediated signaling. FASEB J. 33 (2), 2574–2586. 10.1096/fj.201800920R 30285579

[B20] Hyeon S. Lee H. Yang Y. Jeong W. (2013). Nrf2 deficiency induces oxidative stress and promotes RANKL-induced osteoclast differentiation. Free Radic. Biol. Med. 65, 789–799. 10.1016/j.freeradbiomed.2013.08.005 23954472

[B21] Jackson W. M. Nesti L. J. Tuan R. S. (2012). Concise review: clinical translation of wound healing therapies based on mesenchymal stem cells. Stem Cells Transl. Med. 1 (1), 44–50. 10.5966/sctm.2011-0024 23197639 PMC3727688

[B22] Jang M. K. Yun Y. R. Kim J. H. Park M. H. Jung M. H. (2017). Gomisin N inhibits adipogenesis and prevents high-fat diet-induced obesity. Sci. Rep. 7:40345. 10.1038/srep40345 28067305 PMC5220372

[B23] Kaji K. Katogi R. Azuma Y. Naito A. Inoue J. I. Kudo A. (2001). Tumor necrosis factor alpha-induced osteoclastogenesis requires tumor necrosis factor receptor-associated factor 6. J. Bone Miner. Res. 16 (9), 1593–1599. 10.1359/jbmr.2001.16.9.1593 11547829

[B24] Kim E. J. Lee H. Kim M. H. Yang W. M. (2018). Inhibition of RANKL-stimulated osteoclast differentiation by Schisandra chinensis through down-regulation of NFATc1 and c-fos expression. BMC Complement Altern. Med. 18 (1), 270. 10.1186/s12906-018-2331-5 30285722 PMC6167898

[B25] Lakkakorpi P. T. Vaananen H. K. (1996). Cytoskeletal changes in osteoclasts during the resorption cycle. Microsc. Res. Tech. 33 (2), 171–181. 10.1002/(SICI)1097-0029(19960201)33:2<171::AID-JEMT7>3.0.CO;2-W 8845516

[B26] Lee N. K. Choi Y. G. Baik J. Y. Han S. Y. Jeong D. W. Bae Y. S. (2005). A crucial role for reactive oxygen species in RANKL-induced osteoclast differentiation. Blood 106 (3), 852–859. 10.1182/blood-2004-09-3662 15817678

[B27] Leong P. K. Wong H. S. Chen J. Chan W. M. Leung H. Y. Ko K. M. (2016). Differential Action between Schisandrin A and Schisandrin B in Eliciting an Anti-Inflammatory Action: The Depletion of Reduced Glutathione and the Induction of an Antioxidant Response. PloS One 11 (5), e0155879. 10.1371/journal.pone.0155879 27195753 PMC4873034

[B28] Li J. Lu Y. Wang D. Quan F. Chen X. Sun R. (2019). Schisandrin B prevents ulcerative colitis and colitis-associated-cancer by activating focal adhesion kinase and influence on gut microbiota in an in vivo and in vitro model. Eur. J. Pharmacol. 854, 9–21. 10.1016/j.ejphar.2019.03.059 30951716

[B29] Li Z. Zhu X. Xu R. Wang Y. Hu R. Xu W. (2019). Deacylcynaropicrin Inhibits RANKL-Induced Osteoclastogenesis by Inhibiting NF-kappaB and MAPK and Promoting M2 Polarization of Macrophages. Front. Pharmacol. 10:599:599. 10.3389/fphar.2019.00599 31231214 PMC6567936

[B30] Lin Q. N. Liu Y. D. Guo S. E. Zhou R. Huang Q. Zhang Z. M. (2019). Schisandrin B ameliorates high-glucose-induced vascular endothelial cells injury by regulating the Noxa/Hsp27/NF-kappaB signaling pathway. Biochem. Cell Biol. 97 (6), 681–692. 10.1139/bcb-2018-0321 30817212

[B31] Mou Z. Feng Z. Xu Z. Zhuang F. Zheng X. Li X. (2019). Schisandrin B alleviates diabetic nephropathy through suppressing excessive inflammation and oxidative stress. Biochem. Biophys. Res. Commun. 508 (1), 243–249. 10.1016/j.bbrc.2018.11.128 30477745

[B32] Novack D. V. (2011). Role of NF-kappaB in the skeleton. Cell Res. 21 (1), 169–182. 10.1038/cr.2010.159 21079651 PMC3193402

[B33] Pisani P. Renna M. D. Conversano F. Casciaro E. Di Paola M. Quarta E. (2016). Major osteoporotic fragility fractures: Risk factor updates and societal impact. World J. Orthop. 7 (3), 171–181. 10.5312/wjo.v7.i3.171 27004165 PMC4794536

[B34] Qin J. H. Lin J. R. Ding W. F. Wu W. H. (2019). Schisandrin B Improves the Renal Function of IgA Nephropathy Rats Through Inhibition of the NF-kappaB Signalling Pathway. Inflammation 42 (3), 884–894. 10.1007/s10753-018-0943-z 30519926

[B35] Ran J. Ma C. Xu K. Xu L. He Y. Moqbel S. A. A. (2018). Schisandrin B ameliorated chondrocytes inflammation and osteoarthritis via suppression of NF-kappaB and MAPK signal pathways. Drug Des. Devel. Ther. 12, 1195–1204. 10.2147/DDDT.S162014 PMC5953308 29785089

[B36] Rodan G. A. Martin T. J. (2000). Therapeutic approaches to bone diseases. Science 289 (5484), 1508–1514. 10.1126/science.289.5484.1508 10968781

[B37] Ross F. P. Teitelbaum S. L. (2005). alphavbeta3 and macrophage colony-stimulating factor: partners in osteoclast biology. Immunol. Rev. 208, 88–105. 10.1111/j.0105-2896.2005.00331.x 16313343

[B38] Steinbeck M. J. Appel W. H. Jr. Verhoeven A. J. Karnovsky M. J. (1994). NADPH-oxidase expression and in situ production of superoxide by osteoclasts actively resorbing bone. J. Cell Biol. 126 (3), 765–772. 10.1083/jcb.126.3.765 8045939 PMC2120144

[B39] Sun Y. X. Xu A. H. Yang Y. Li J. (2015). Role of Nrf2 in bone metabolism. J. BioMed. Sci. 22, 101. 10.1186/s12929-015-0212-5 26511009 PMC4625735

[B40] Takanche J. S. Kim J. E. Han S. H. Yi H. K. (2020). Effect of gomisin A on osteoblast differentiation in high glucose-mediated oxidative stress. Phytomedicine 66:153107. 10.1016/j.phymed.2019.153107 31790903

[B41] Weaver M. J. Miller M. A. Vrahas M. S. (2010). The orthopaedic implications of diphosphonate therapy. J. Am. Acad. Orthop. Surg. 18 (6), 367–374. 10.5435/00124635-201006000-00009 20511442

[B42] Yin Z. Zhu W. Wu Q. Zhang Q. Guo S. Liu T. (2019). Glycyrrhizic acid suppresses osteoclast differentiation and postmenopausal osteoporosis by modulating the NF-kappaB, ERK, and JNK signaling pathways. Eur. J. Pharmacol. 859:172550. 10.1016/j.ejphar.2019.172550 31323222

[B43] You S. Qian J. Wu G. Qian Y. Wang Z. Chen T. (2019). Schizandrin B attenuates angiotensin II induced endothelial to mesenchymal transition in vascular endothelium by suppressing NF-kappaB activation. Phytomedicine 62:152955. 10.1016/j.phymed.2019.152955 31146168

[B44] Zalli D. Neff L. Nagano K. Shin N. Y. Witke W. Gori F. (2016). The Actin-Binding Protein Cofilin and Its Interaction With Cortactin Are Required for Podosome Patterning in Osteoclasts and Bone Resorption In Vivo and In Vitro. J. Bone Miner. Res. 31 (9), 1701–1712. 10.1002/jbmr.2851 27064822 PMC5070801

[B45] Zhao X. Xiang Y. Cai C. Zhou A. Zhu N. Zeng C. (2018). Schisandrin B protects against myocardial ischemia/reperfusion injury via the PI3K/Akt pathway in rats. Mol. Med. Rep. 17 (1), 556–561. 10.3892/mmr.2017.7926 29115607

[B46] Zhu N. Cai C. Zhou A. Zhao X. Xiang Y. Zeng C. (2017). Schisandrin B Prevents Hind Limb from Ischemia-Reperfusion-Induced Oxidative Stress and Inflammation via MAPK/NF-kappaB Pathways in Rats. BioMed. Res. Int. 2017:4237973. 10.1155/2017/4237973 28706944 PMC5494555

