# Supplementary figures and images for: Schisandrin B Inhibits Osteoclastogenesis and Protects Against Ovariectomy-Induced Bone Loss
Source: Front Pharmacol. 2020 Jul 31;11:1175. doi: 10.3389/fphar.2020.01175 (PMC7413103; doi:10.3389/fphar.2020.01175)

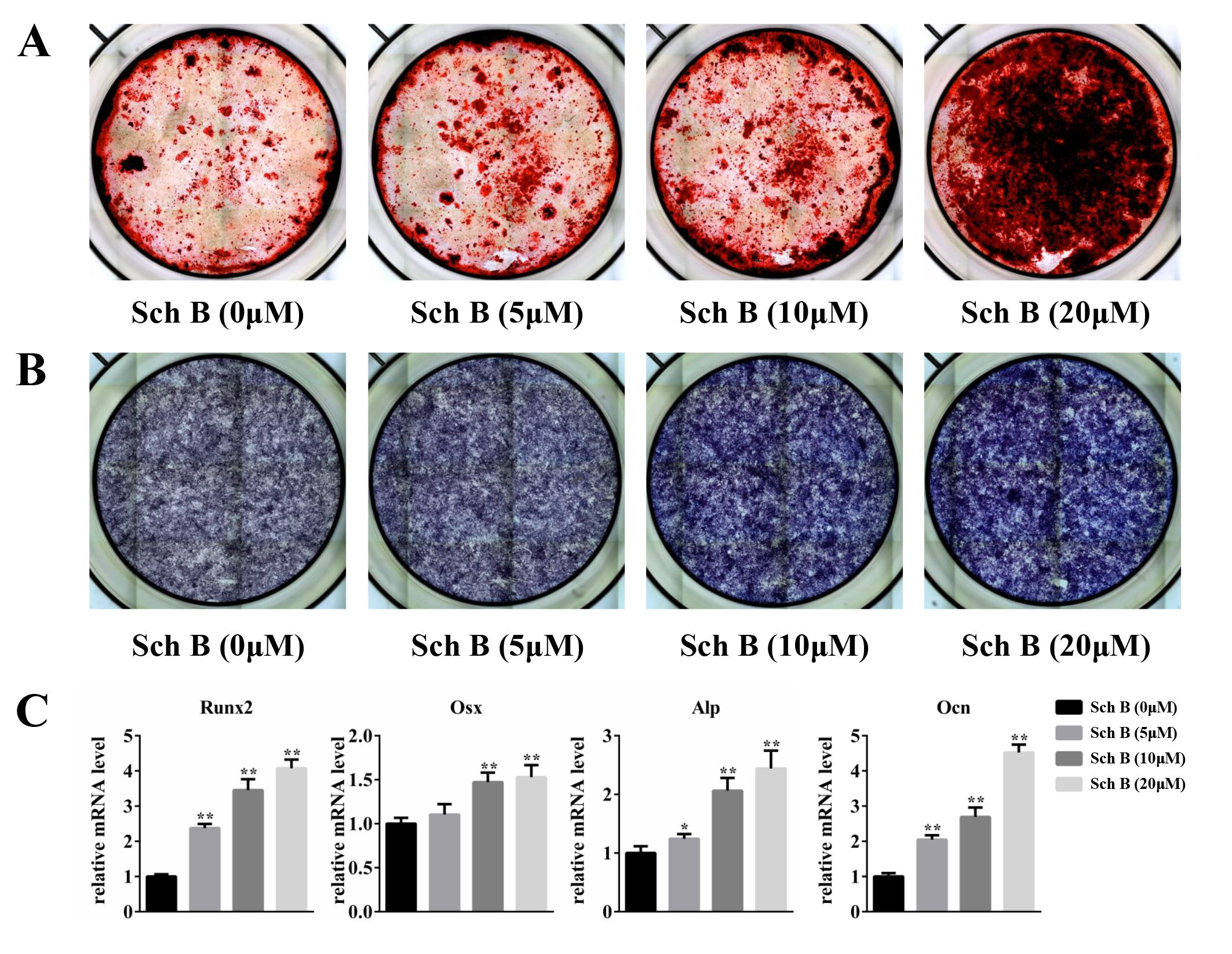

Supplement: Supplementary Figure 1 — The plant sample of Schisandrin B promotes osteoblast differentiation. BMSCs were cultured in osteogenic medium and treated with different concentrations of Schisandrin B as indicated. (A) representative Alizarin staining pictures, (B) representative ALP staining results, (C) mRNA levels of Runx2, Osx, Alp, and Ocn. *Plt;0.05, **P<0.01 versus the vehicle. [file Image_1.tif]

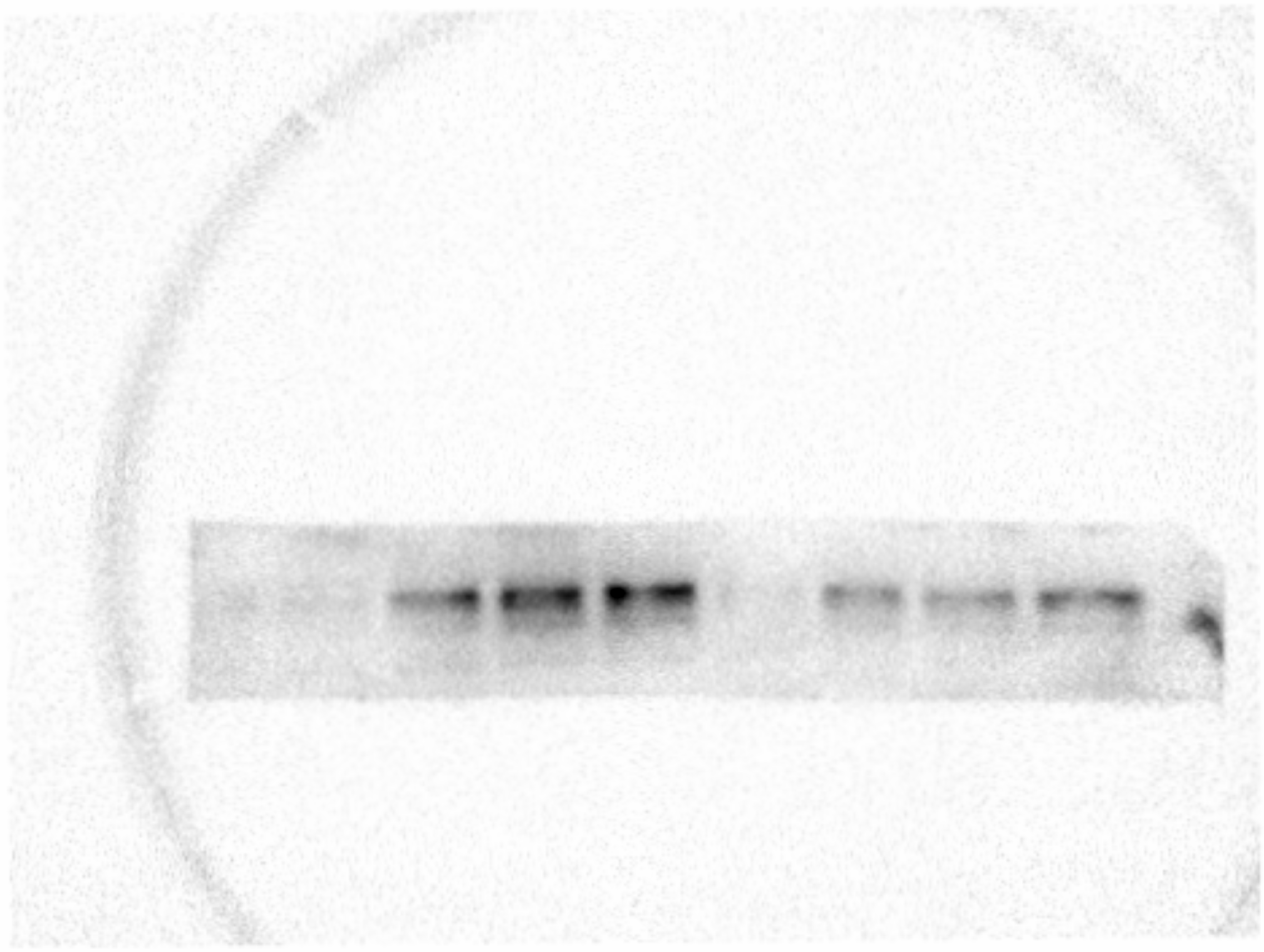

Supplement: Supplementary file 2 [file Presentation_1.zip › Figure5B.c-FOS.tif]

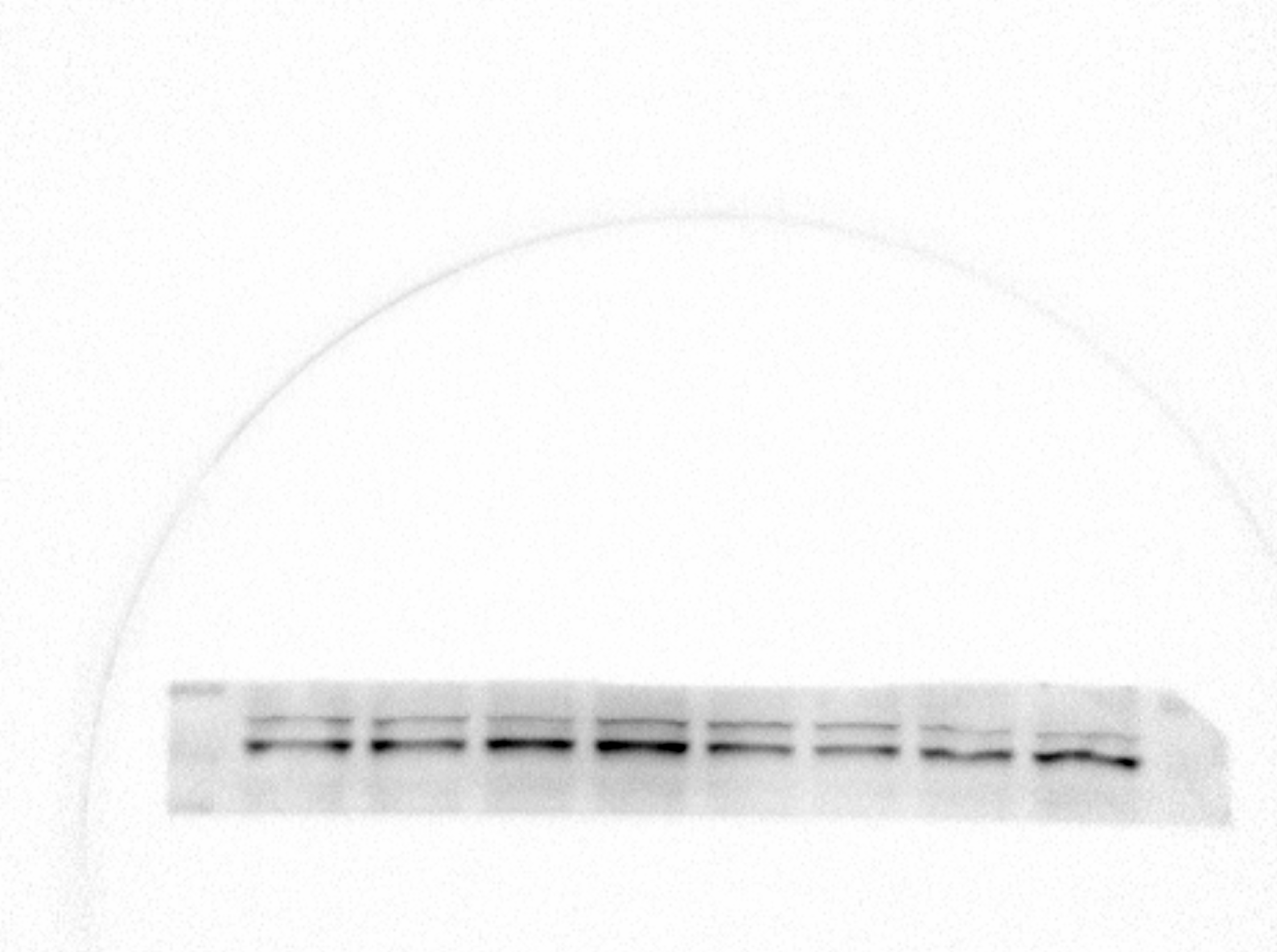

Supplement: Supplementary file 2 [file Presentation_1.zip › Figure5B.MMP9.tif]

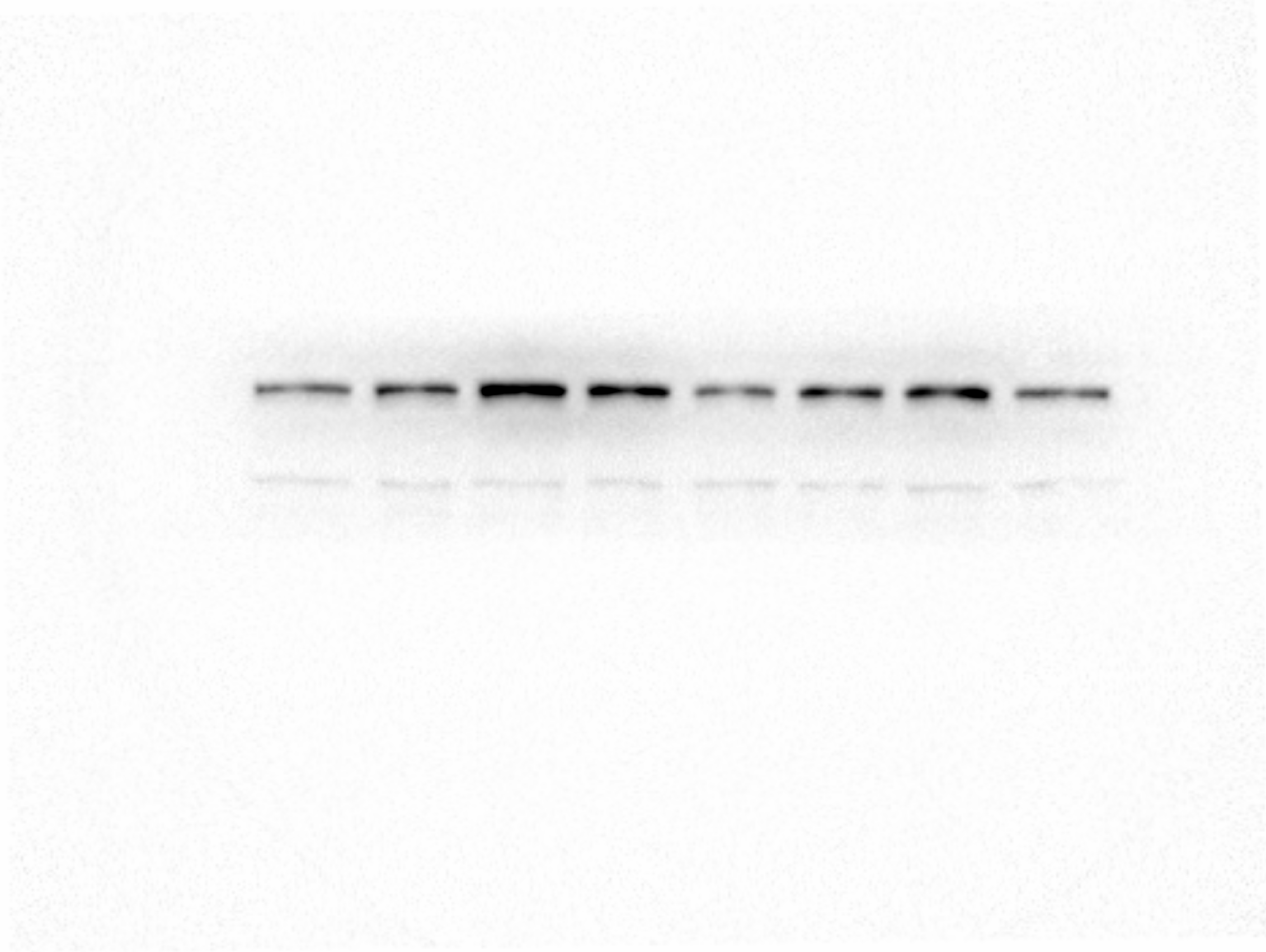

Supplement: Supplementary file 2 [file Presentation_1.zip › Figure5B.NFATc1.tif]

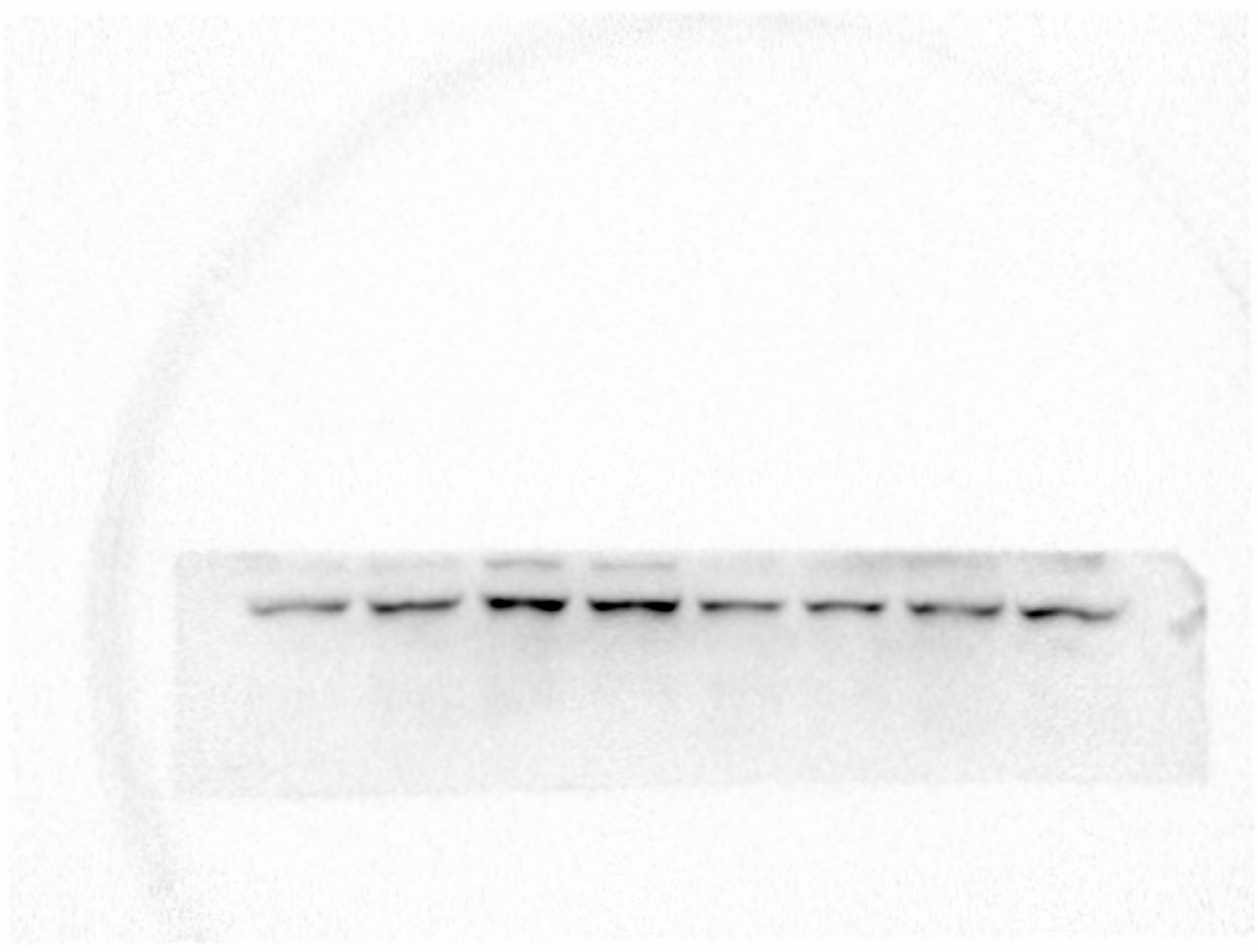

Supplement: Supplementary file 2 [file Presentation_1.zip › Figure5B.TRAP.tif]

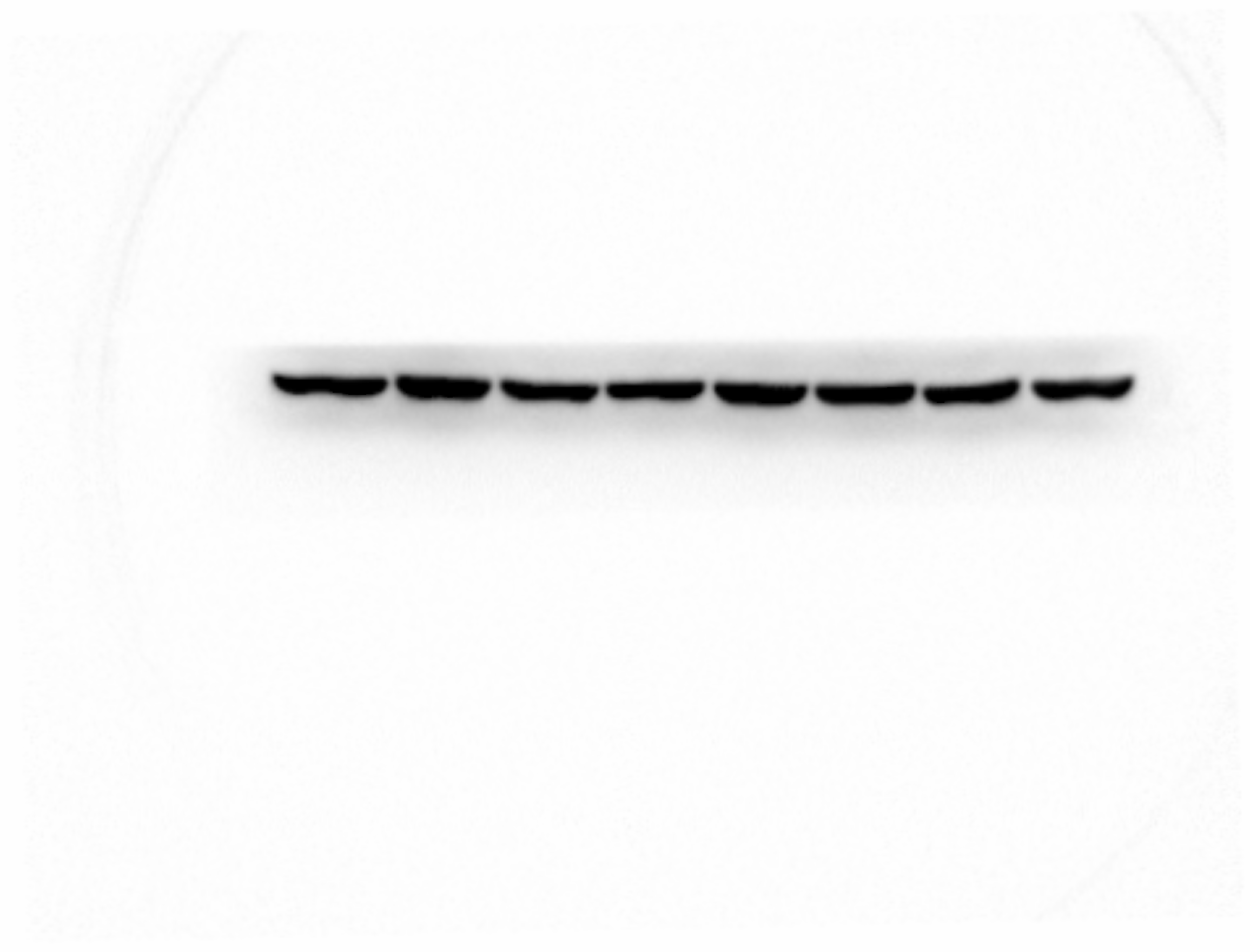

Supplement: Supplementary file 2 [file Presentation_1.zip › Figure5B.β-actin.tif]

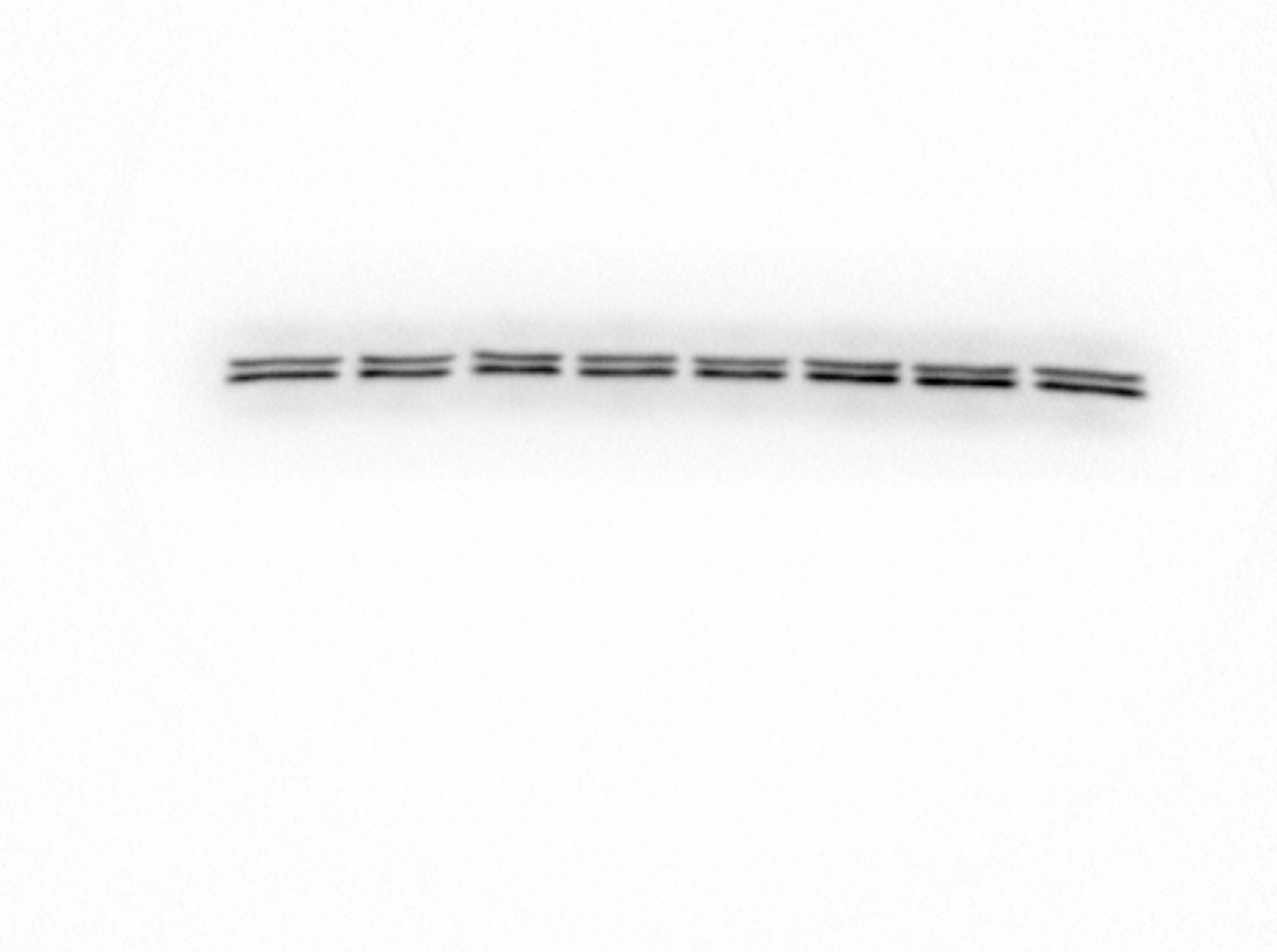

Supplement: Supplementary file 2 [file Presentation_1.zip › Figure6A.ERK.tif]

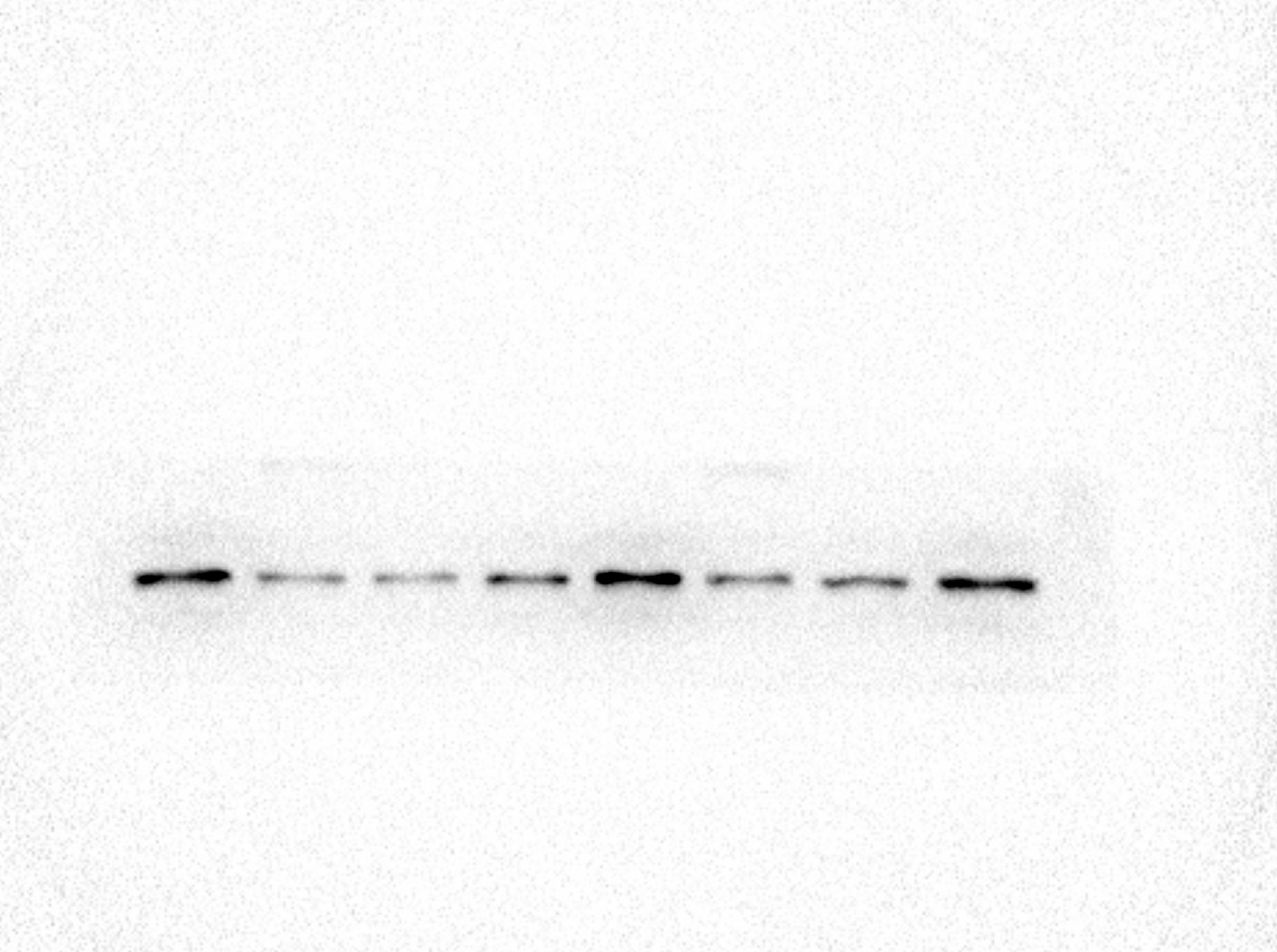

Supplement: Supplementary file 2 [file Presentation_1.zip › Figure6A.IκBα.tif]

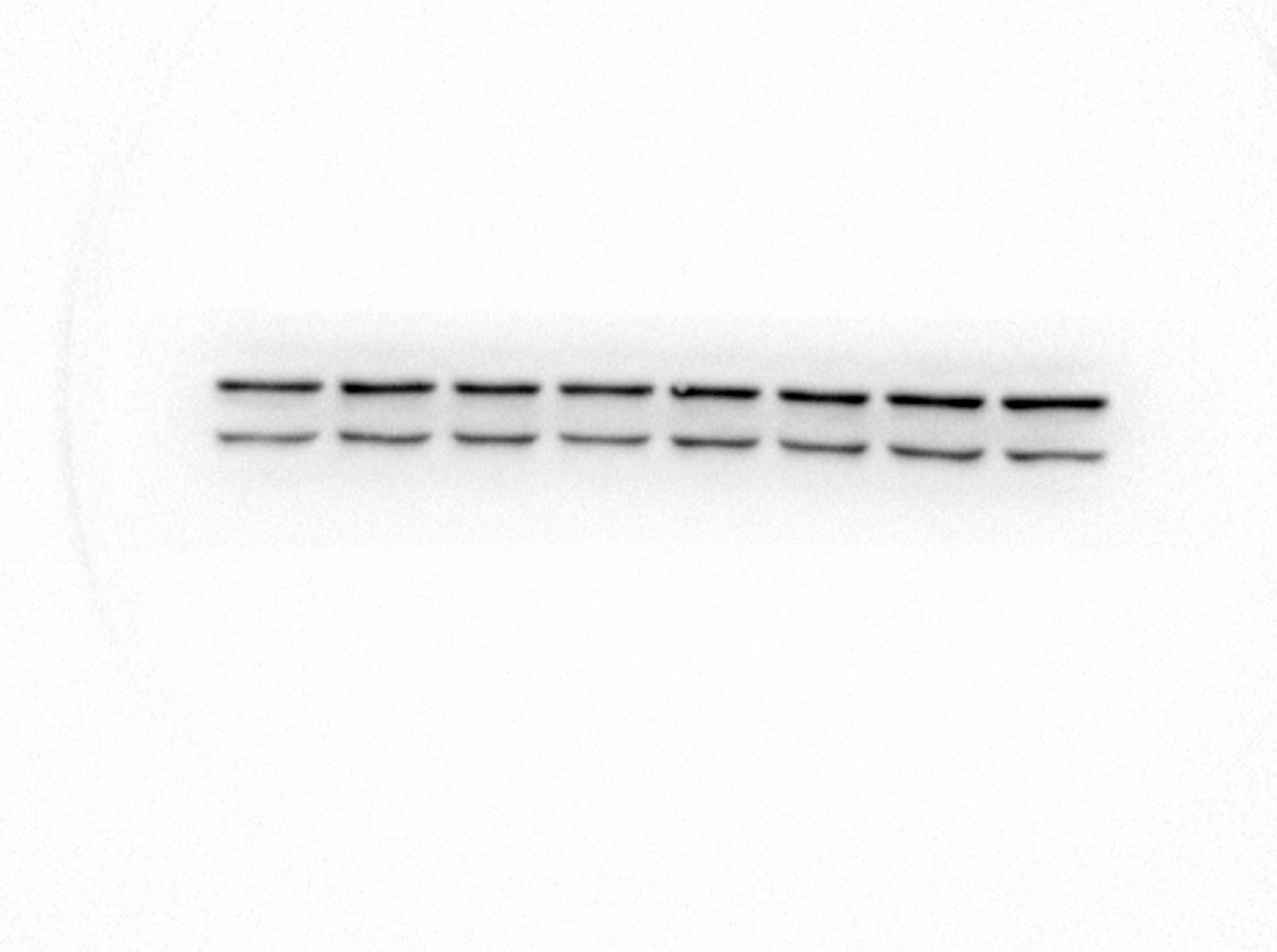

Supplement: Supplementary file 2 [file Presentation_1.zip › Figure6A.JNK.tif]

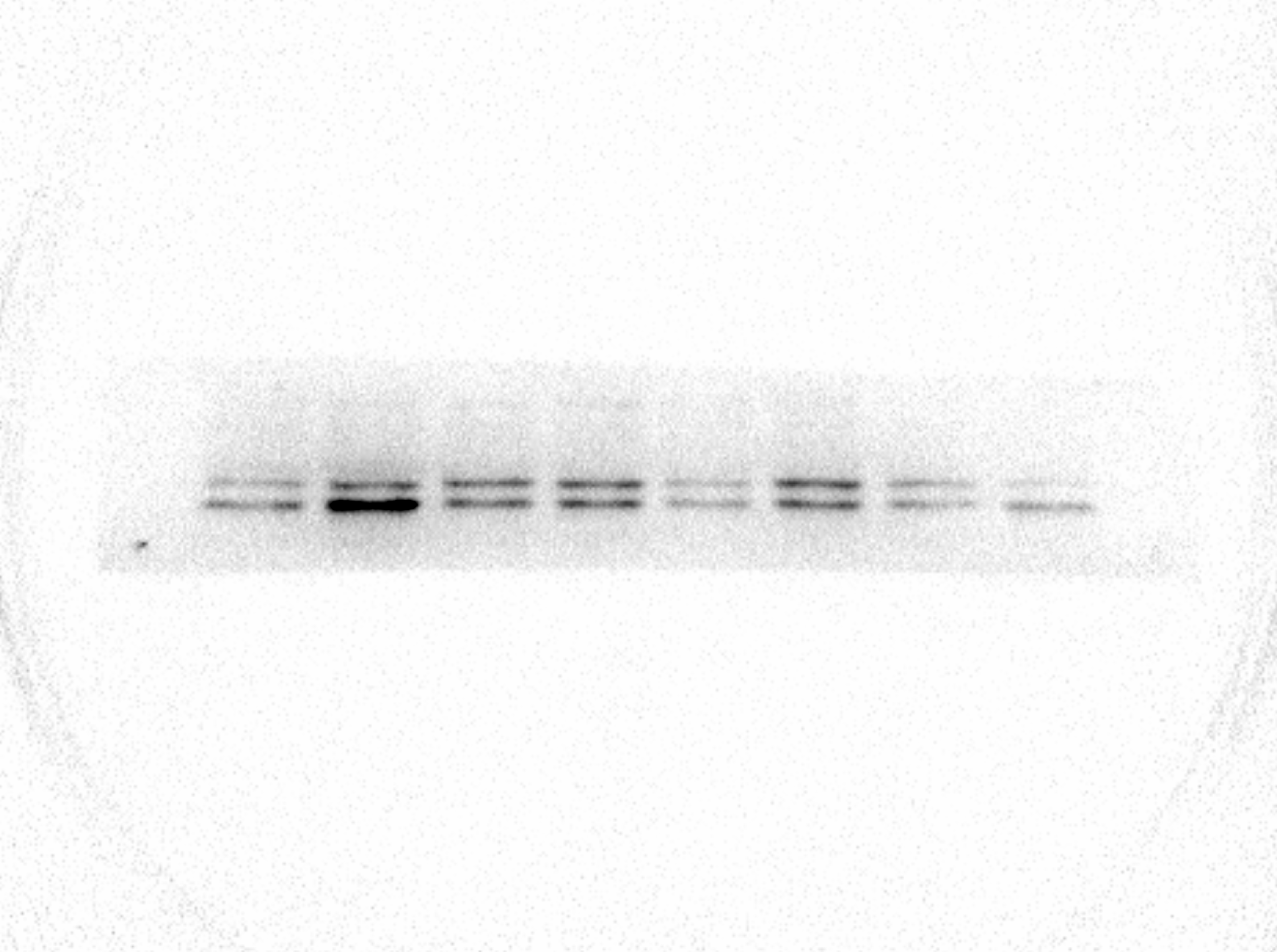

Supplement: Supplementary file 2 [file Presentation_1.zip › Figure6A.p-ERK.tif]

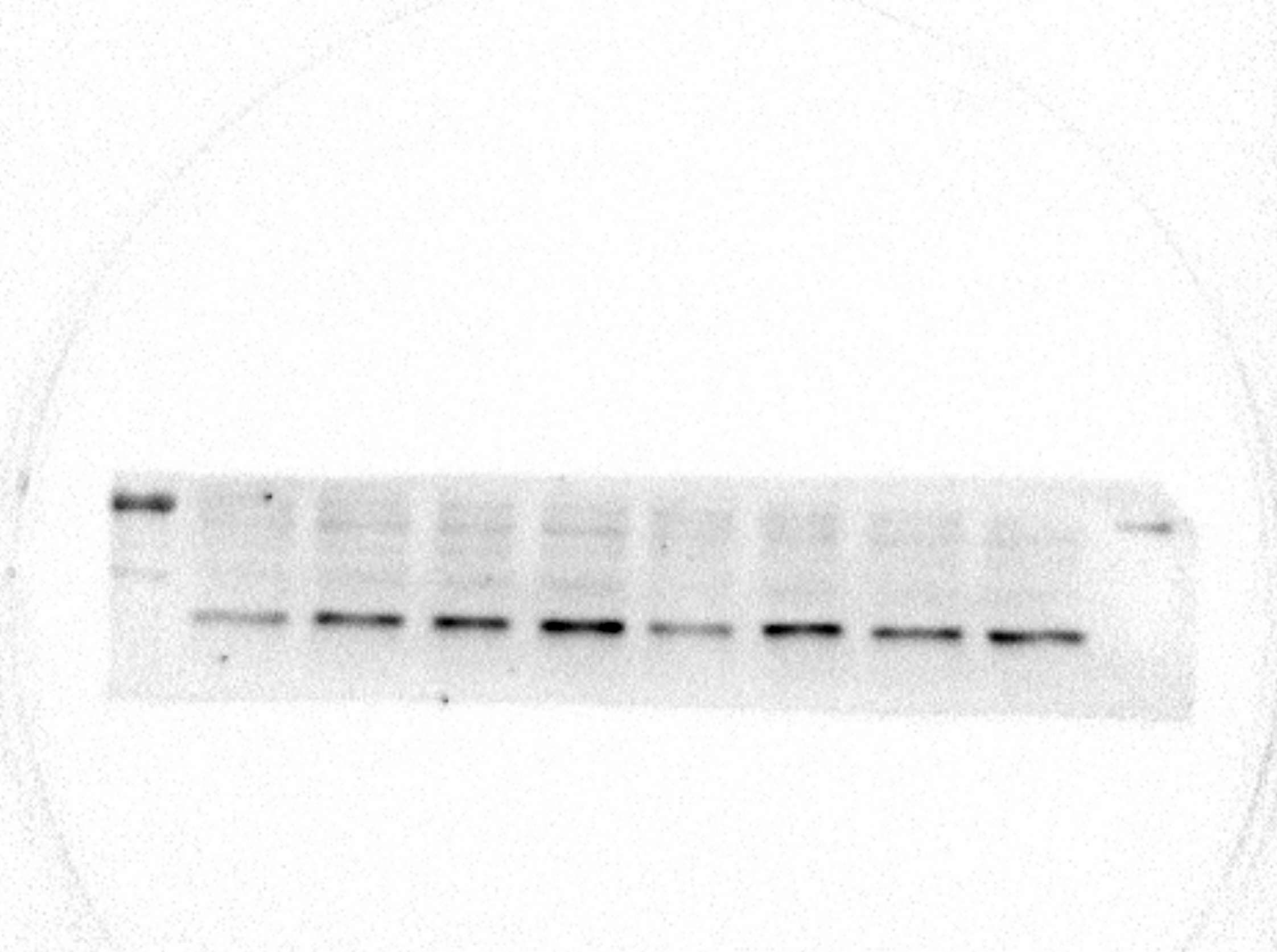

Supplement: Supplementary file 2 [file Presentation_1.zip › Figure6A.p-IκBα.tif]

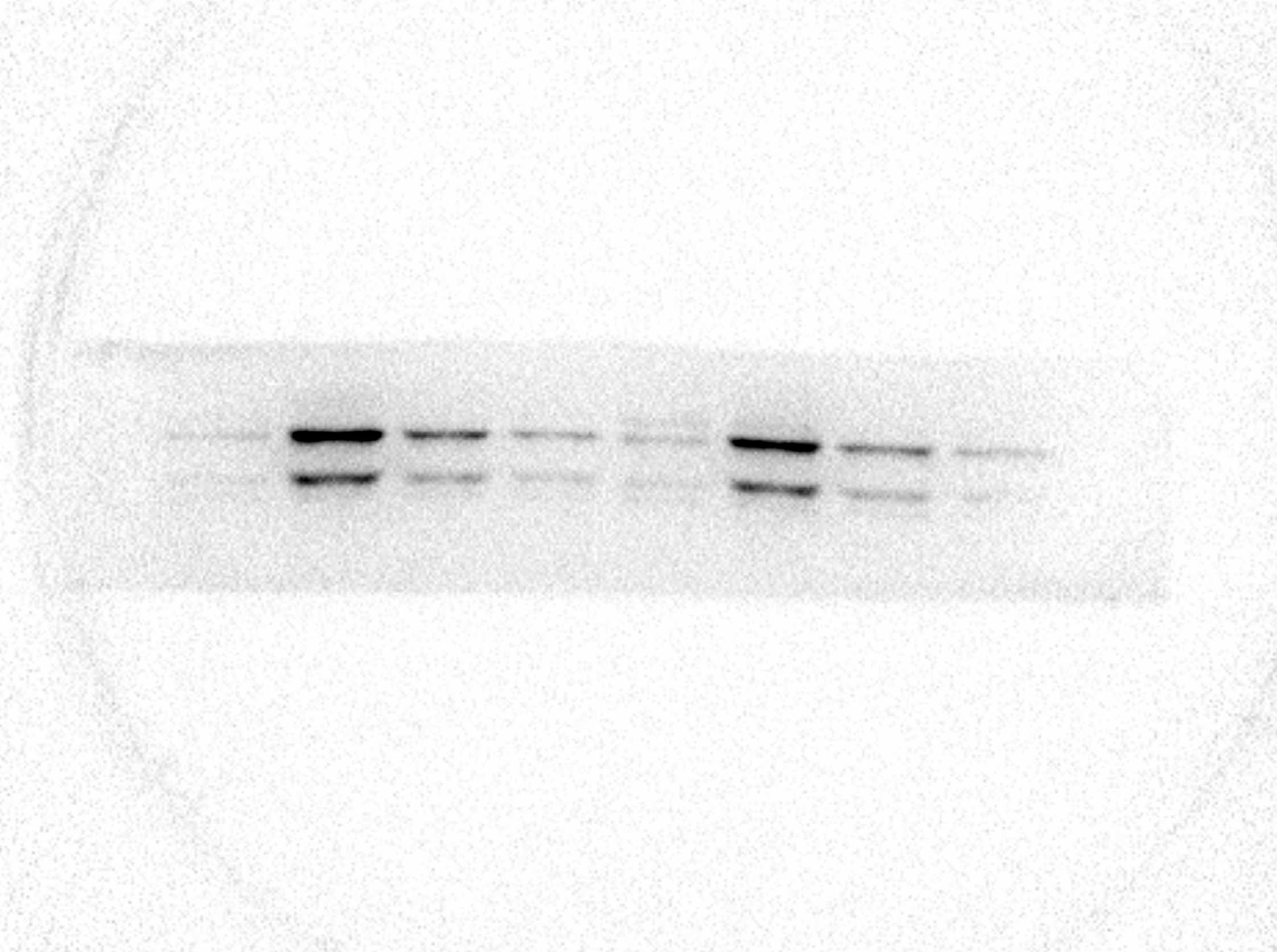

Supplement: Supplementary file 2 [file Presentation_1.zip › Figure6A.p-JNK.tif]

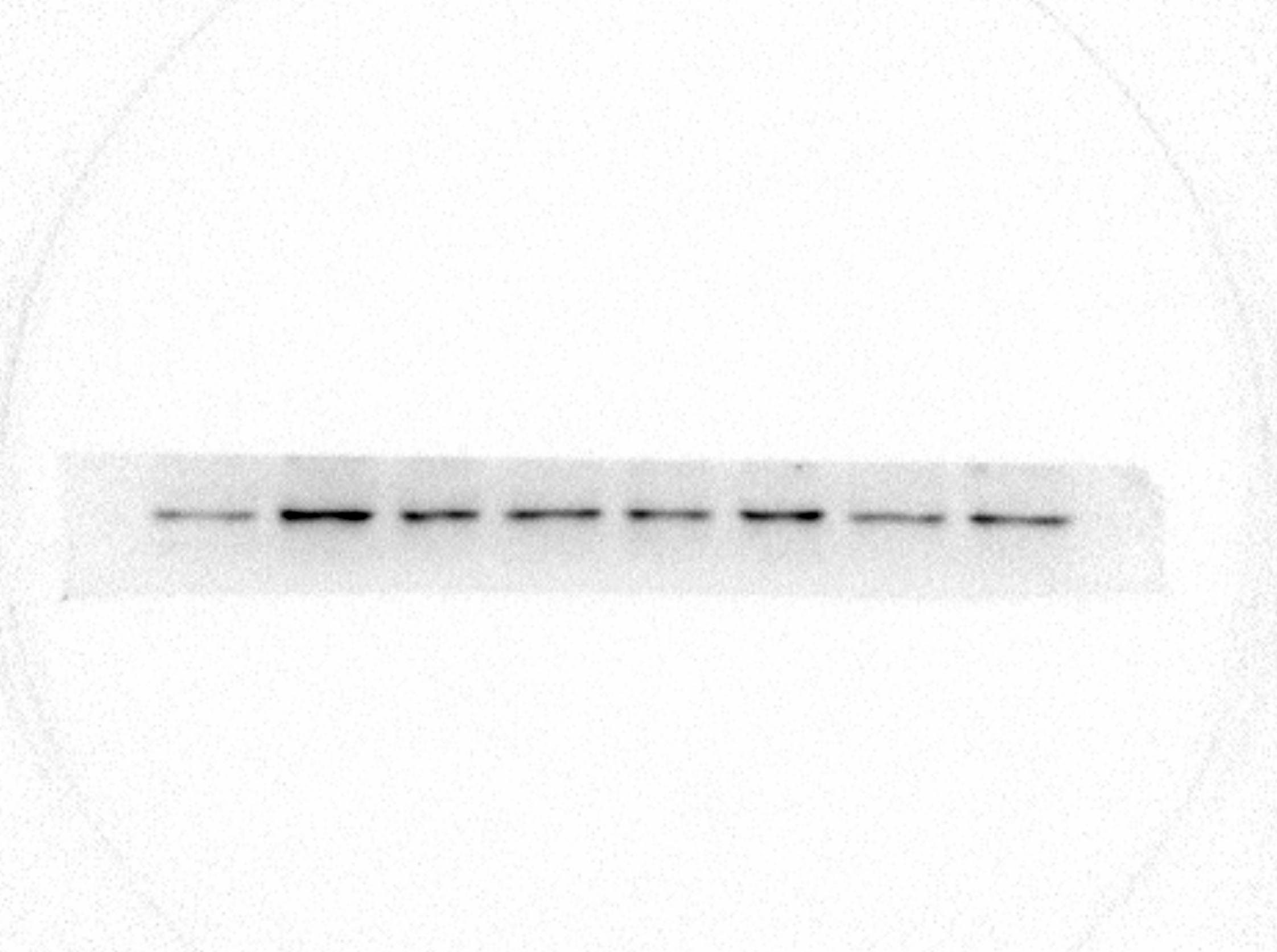

Supplement: Supplementary file 2 [file Presentation_1.zip › Figure6A.p-P38.tif]

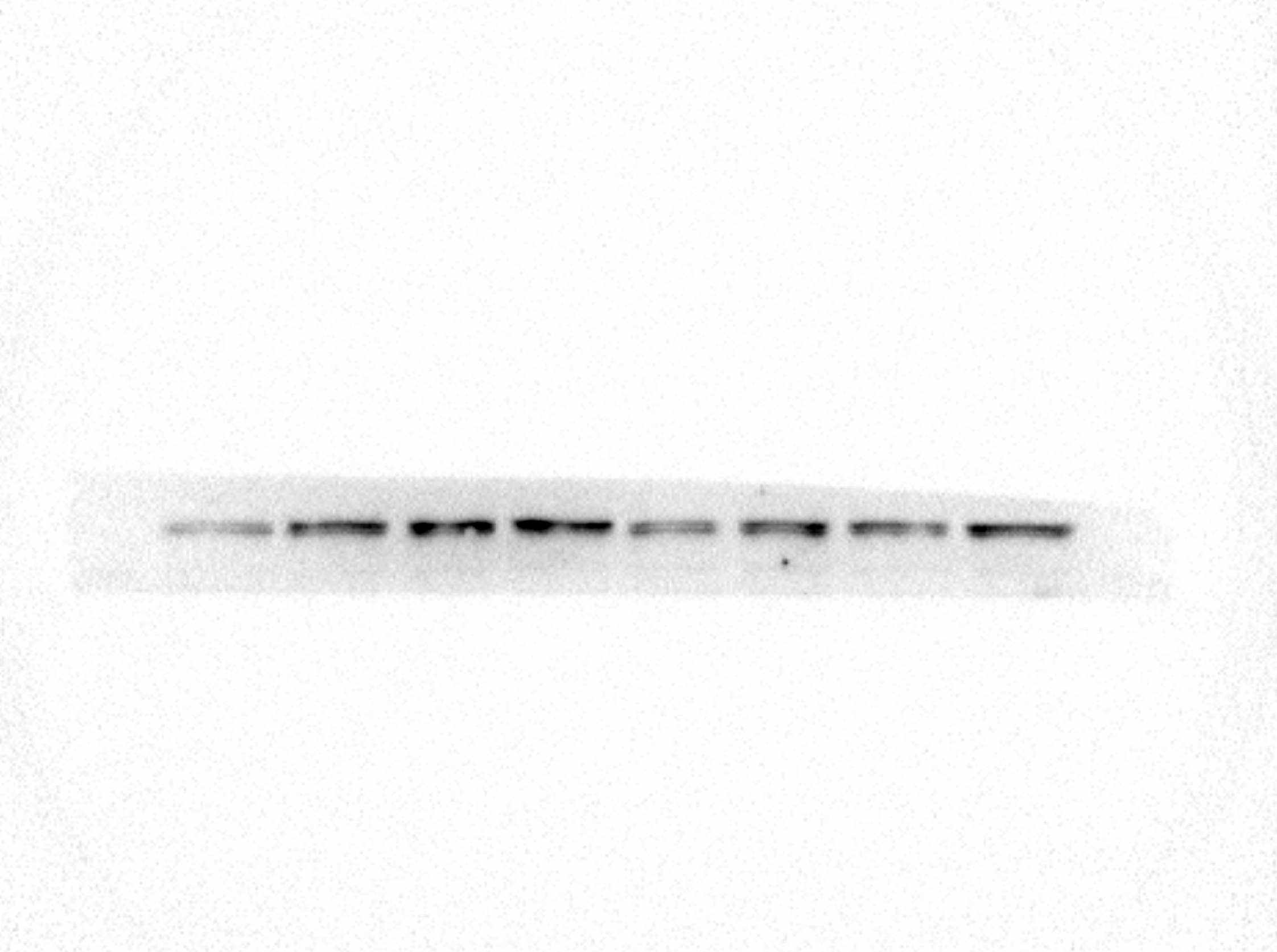

Supplement: Supplementary file 2 [file Presentation_1.zip › Figure6A.p-P65.tif]

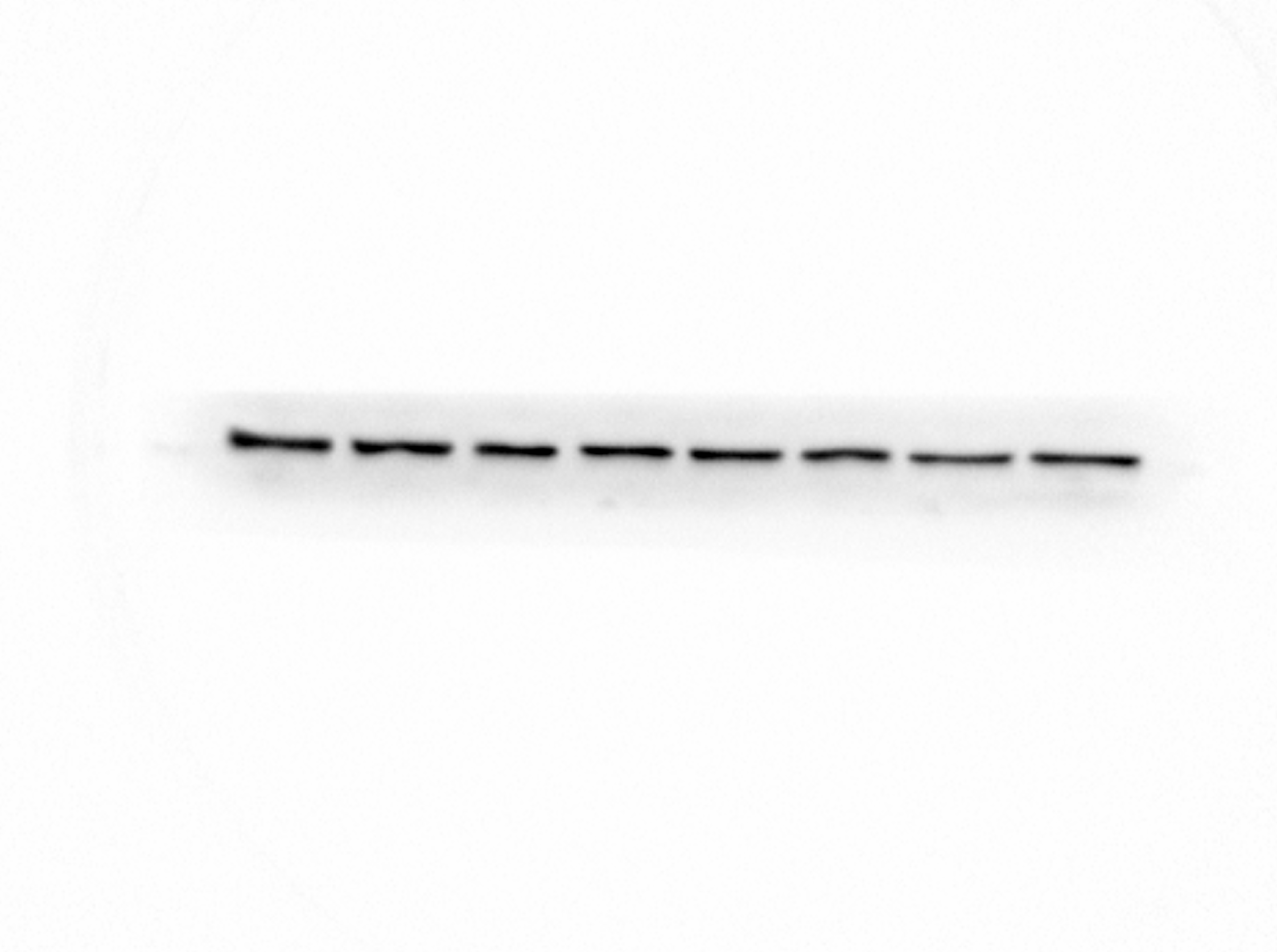

Supplement: Supplementary file 2 [file Presentation_1.zip › Figure6A.P38.tif]

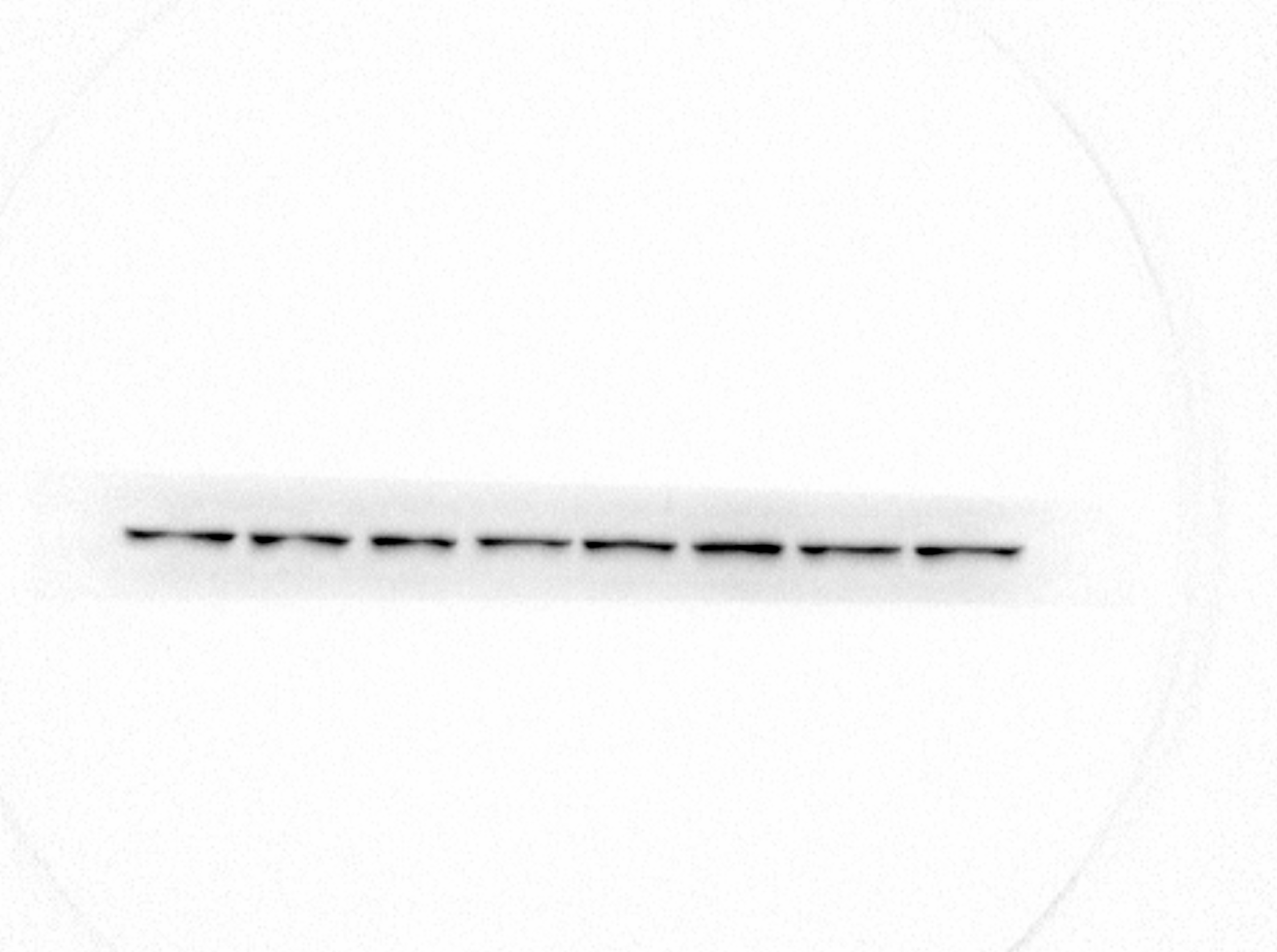

Supplement: Supplementary file 2 [file Presentation_1.zip › Figure6A.P65.tif]

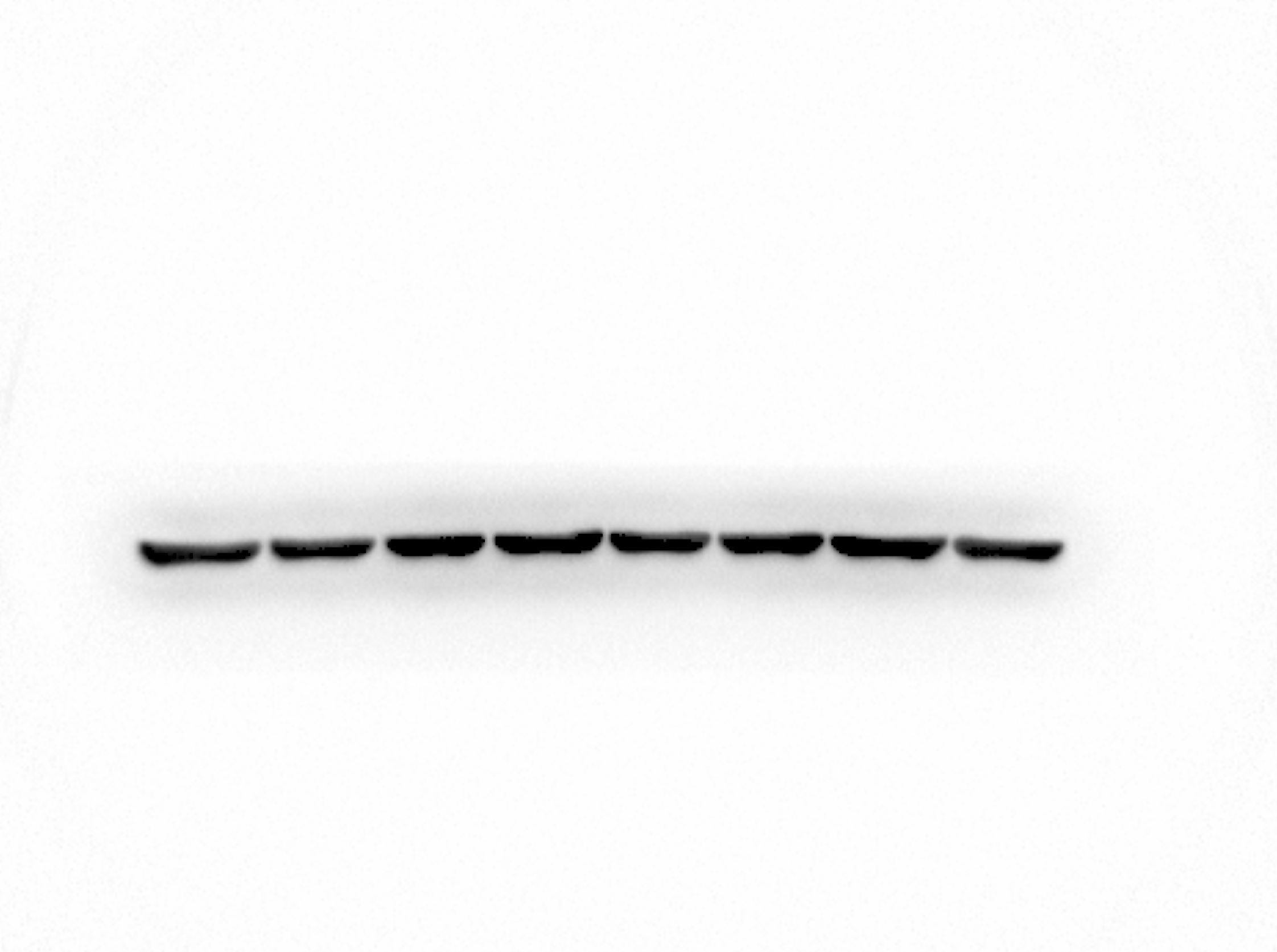

Supplement: Supplementary file 2 [file Presentation_1.zip › Figure6A.β-actin1.tif]

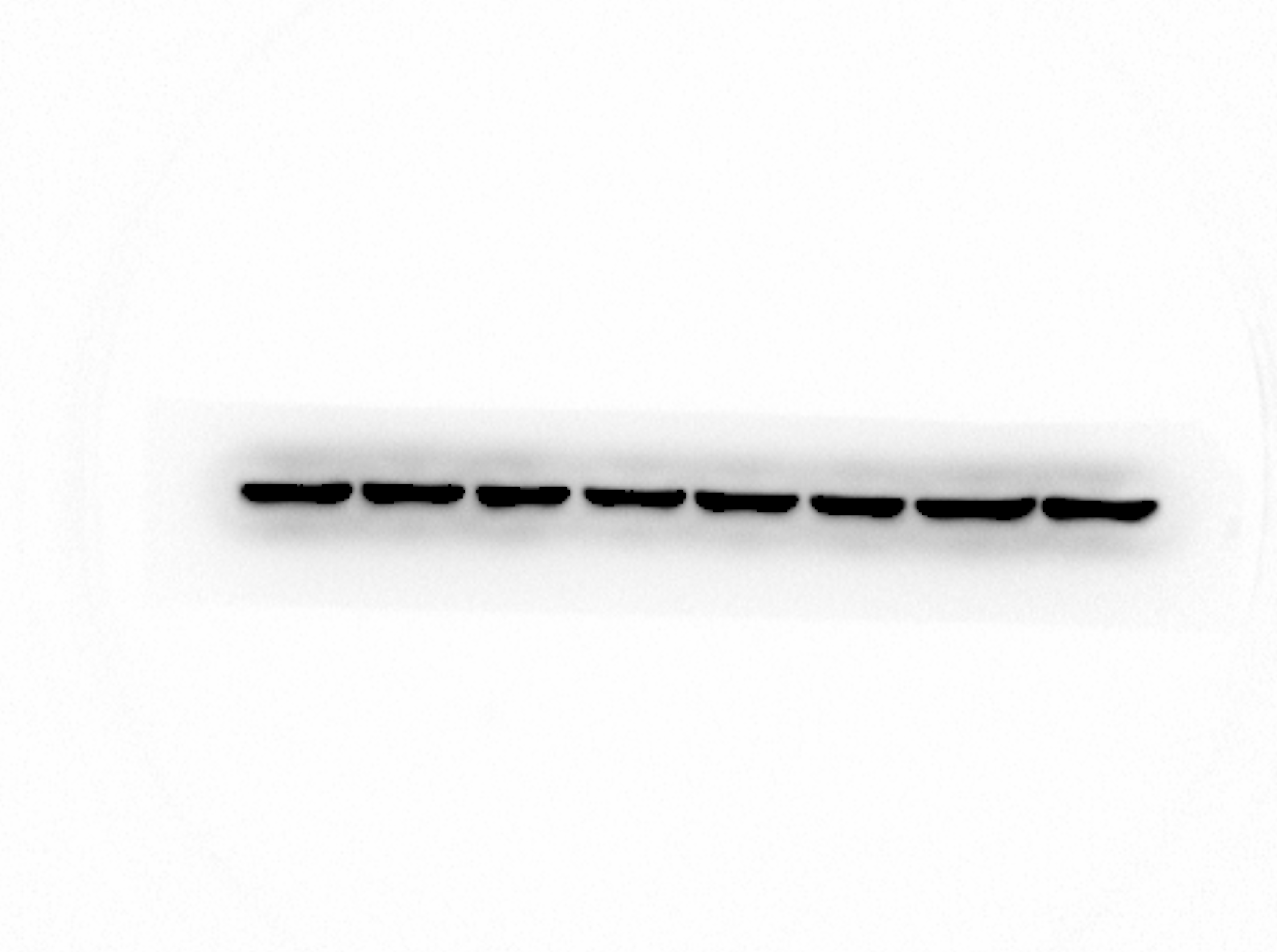

Supplement: Supplementary file 2 [file Presentation_1.zip › Figure6A.β-actin2.tif]

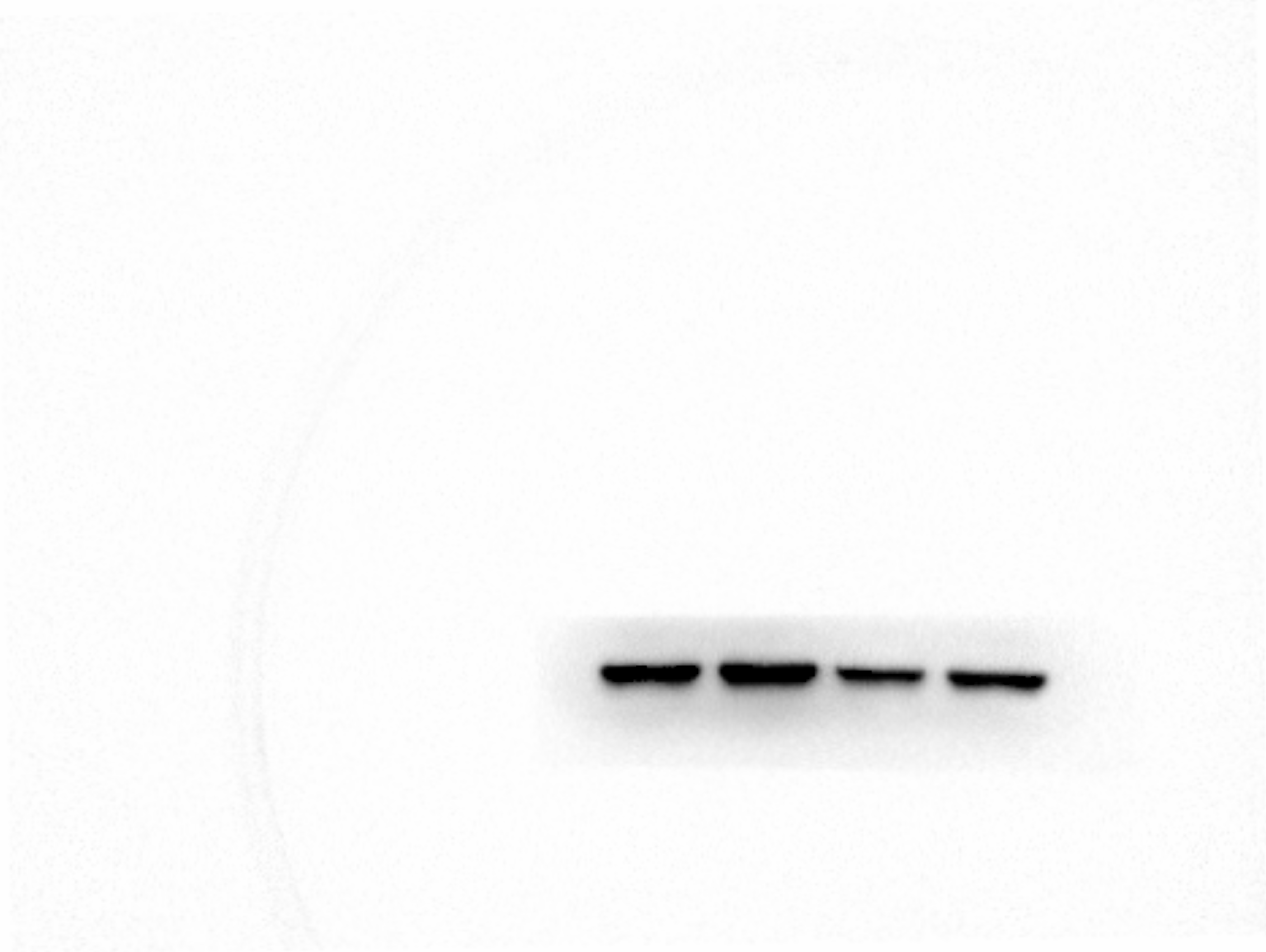

Supplement: Supplementary file 2 [file Presentation_1.zip › Figure7B.HO1.tif]

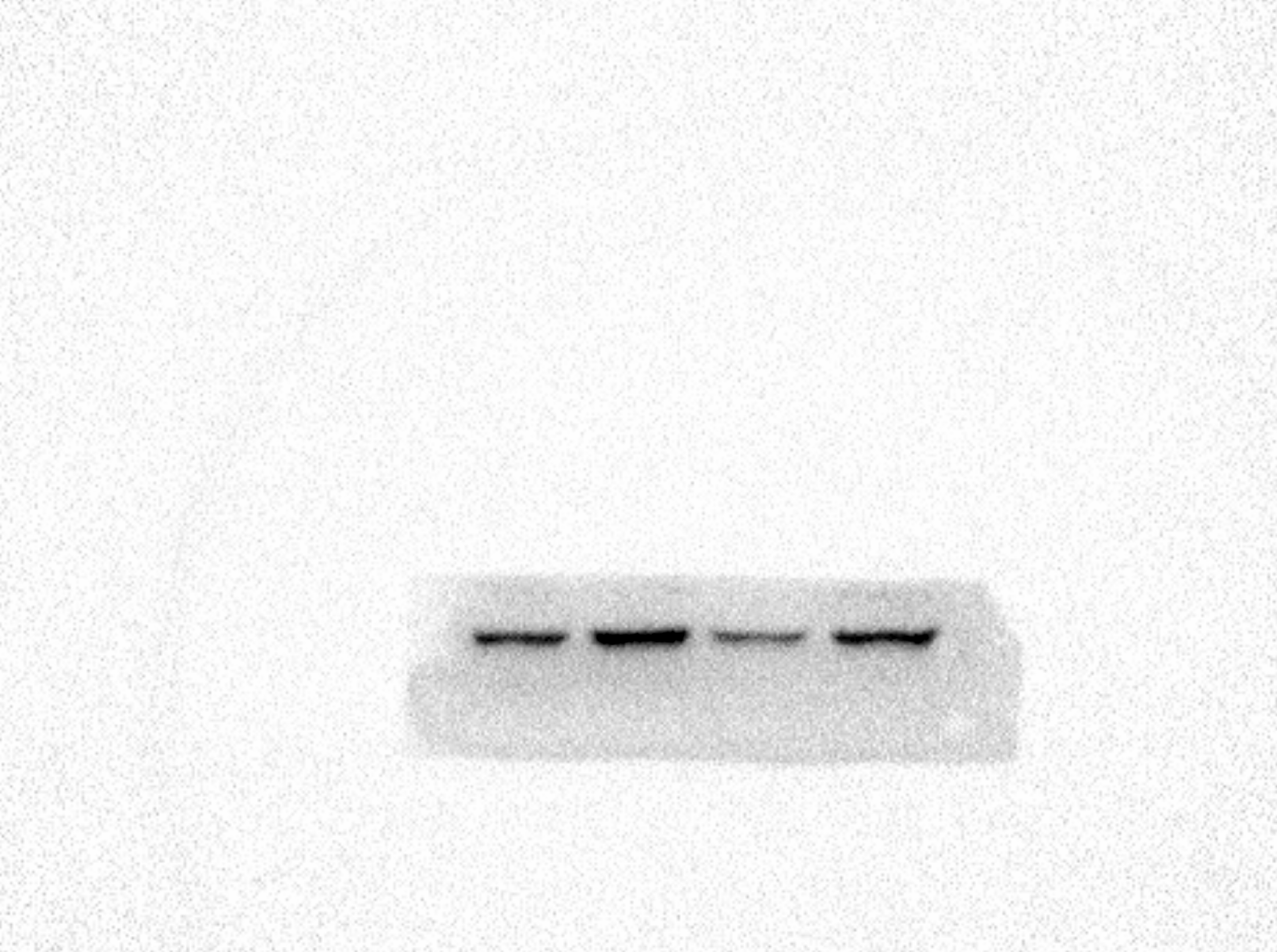

Supplement: Supplementary file 2 [file Presentation_1.zip › Figure7B.NQO1.tif]

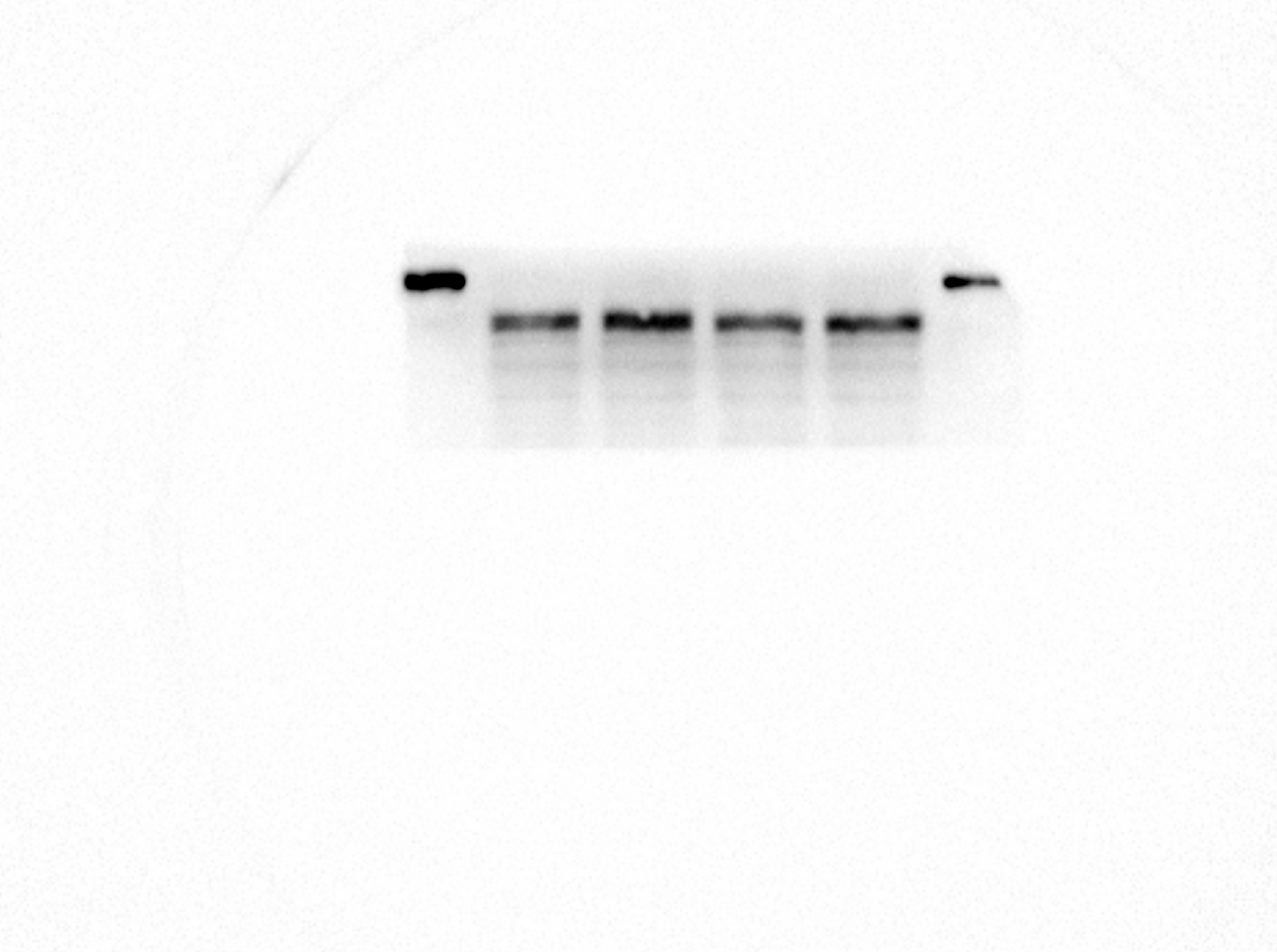

Supplement: Supplementary file 2 [file Presentation_1.zip › Figure7B.NRF2.tif]

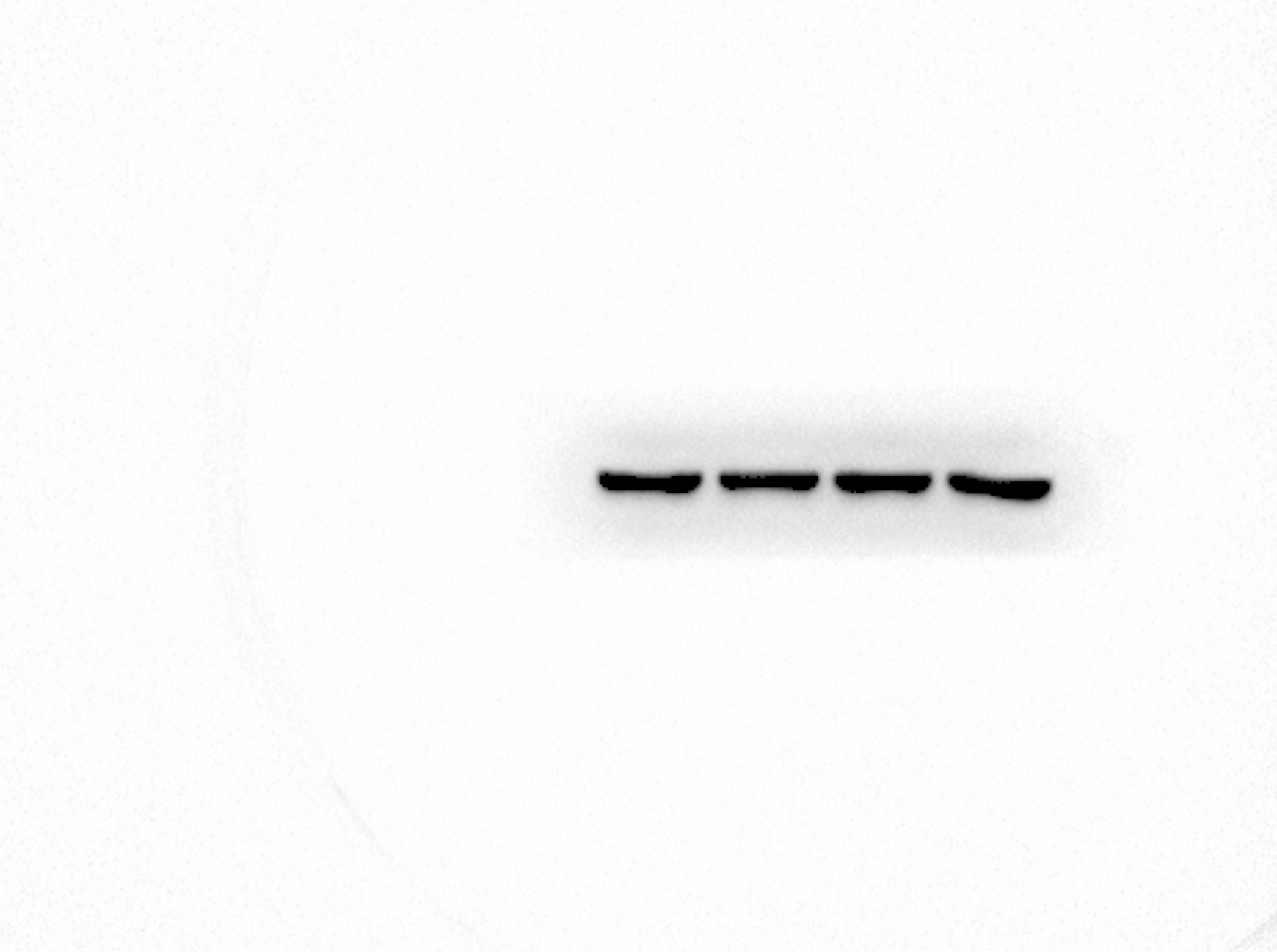

Supplement: Supplementary file 2 [file Presentation_1.zip › Figure7B.β-actin.tif]
